# The Mercury Resistance Operon: From an Origin in a Geothermal Environment to an Efficient Detoxification Machine

**DOI:** 10.3389/fmicb.2012.00349

**Published:** 2012-10-08

**Authors:** Eric S. Boyd, Tamar Barkay

**Affiliations:** ^1^Department of Chemistry and Biochemistry, Montana State UniversityBozeman, MT, USA; ^2^Department of Biochemistry and Microbiology, Rutgers UniversityNew Brunswick, NJ, USA

**Keywords:** *merA*, mercuric reductase, trait evolution, diversity, genomics, gene loss, lateral gene transfer, operon evolution

## Abstract

Mercuric mercury (Hg[II]) is a highly toxic and mobile element that is likely to have had a pronounced and adverse effect on biology since Earth’s oxygenation ∼2.4 billion years ago due to its high affinity for protein sulfhydryl groups, which upon binding destabilize protein structure and decrease enzyme activity, resulting in a decreased organismal fitness. The central enzyme in the microbial mercury detoxification system is the mercuric reductase (MerA) protein, which catalyzes the reduction of Hg(II) to volatile Hg(0). In addition to MerA, *mer* operons encode for proteins involved in regulation, Hg binding, and organomercury degradation. Mer-mediated approaches have had broad applications in the bioremediation of mercury-contaminated environments and industrial waste streams. Here, we examine the composition of 272 individual *mer* operons and quantitatively map the distribution of *mer*-encoded functions on both taxonomic SSU rRNA gene and MerA phylogenies. The results indicate an origin and early evolution of MerA among thermophilic bacteria and an overall increase in the complexity of *mer* operons through evolutionary time, suggesting continual gene recruitment and evolution leading to an improved efficiency and functional potential of the Mer detoxification system. Consistent with a positive relationship between the evolutionary history and topology of MerA and SSU rRNA gene phylogenies (Mantel *R* = 0.81, *p *< 0.01), the distribution of the majority of *mer* functions, when mapped on these phylograms, indicates an overall tendency to inherit *mer*-encoded functions through vertical descent. However, individual *mer* functions display evidence of a variable degree of vertical inheritance, with several genes exhibiting strong evidence for acquisition via lateral gene transfer and/or gene loss. Collectively, these data suggest that (i) *mer* has evolved from a simple system in geothermal environments to a widely distributed and more complex and efficient detoxification system, and (ii) *merA* is a suitable biomarker for examining the functional diversity of Hg detoxification and for predicting the composition of *mer* operons in natural environments.

## Introduction

Mercury (Hg) is the most toxic heavy metal due to its high affinity for the sulfhydryl ligands in amino acids, which upon binding, leads to alteration in protein structure, and often a loss of function (Nies, 2003). Two unique aspects of Hg geochemistry place it high on the list of potent environmental contaminants: its global distribution (Pirrone et al., 2010) and the possibility of it being converted to more toxic methylated forms (Lin et al., 2012). Methylated forms of mercury (MeHg) are produced in the environment by anaerobic bacteria and the MeHg that is produced is often bioaccumulated and biomagnified in aquatic (Watras et al., 1998; Boyd et al., 2009) and terrestrial (Rimmer et al., 2010) food chains, thereby posing severe consequences to human and environmental health (Clarkson and Magos, 2006). Moreover, atmospheric deposition of Hg, coupled with methylation and biomagnification, lead to accumulation of MeHg in biota far removed from known sources of Hg (e.g., polar-regions; Dietz et al., 2009).

**Figure 1:**
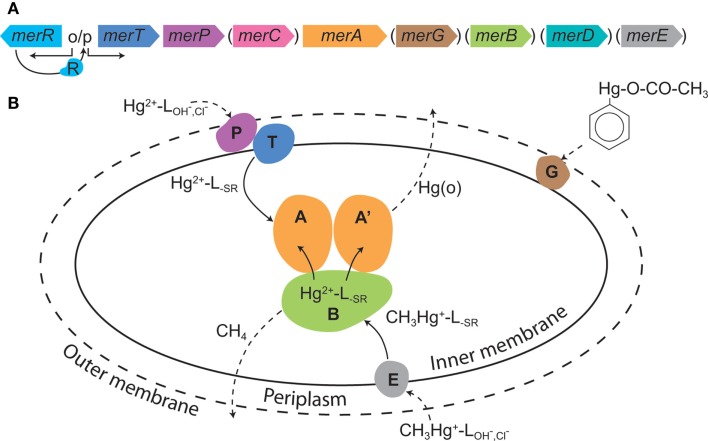
**The *mer* system**. **(A)** A generic *mer* operon with genes in parentheses depicting those that are present in some, but not the majority of, operons. **(B)** The cellular *mer*-encoded mercury detoxification mechanisms. The outer cell wall is depicted by a broken line illustrating that not all microbes have an outer membrane; broken line arrows depict diffusion; solid line arrows indicate transport or transformations; L = ligand with subscripts denoting the ligand type. The colors of various Mer proteins correspond with the colors of the genes that encode for these proteins in **(A)**. Reproduced with permission from Lin et al. (2012).

Some aerobic Bacteria and Archaea have evolved resistance mechanisms that function to degrade organomercury compounds and to reduce the local concentration of inorganic Hg (Hg[II]) by reduction to gaseous Hg(0), effectively partitioning Hg(0) to the gaseous phase, and allowing for microbial growth (Barkay et al., 2003; Lin et al., 2012). The Hg resistance (*mer*) system is encoded for by the *mer* operon that consists of the homodimeric flavin-dependent disulfide oxidoreductase enzyme mercuric reductase (MerA) and which may also encode for organomercury lyase (MerB), a periplasmic Hg(II) scavenging protein (MerP), one or more inner membrane spanning proteins (MerT, MerC, MerE, MerF, MerG) that transport Hg(II) to the cytoplasm where it is reduced by MerA, and one or two regulatory proteins (MerR, MerD; Figure 1). The overall expression of *mer* is regulated by MerR, acting as a transcriptional repressor or activator in the absence and presence of Hg(II), respectively. In addition, several proteobacteria encode for MerD which functions to down regulate operon expression (Barkay et al., 2003; Lin et al., 2012).

About 1–10% of cultured heterotrophic, aerobic microbes from various environments possess *mer* systems (Barkay, 1987), alluding to the ubiquity of Hg(II) in the environment, and the high selective pressure that this imposes on microbiota to detoxify their local environment. Mercury resistant microorganisms are often enriched to even higher abundances in Hg contaminated environments (Barkay, 1987; Osborn et al., 1997), where their activities enhance conversion of MeHg (Schaefer et al., 2004) and Hg(II) to Hg(0) (Kritee et al., 2007). Furthermore, bacteria that resist Hg via the *mer* system have been utilized to effectively remove Hg from chlor-alkali bioreactor wastewater, yielding water that is suitable for disposal to municipal waste streams (Wagner-Döbler, 2003). The comprehensive understanding of *mer* and the functions that it encodes have facilitated the bioengineering of molecules, cells, and plants that are efficient at sorbing and/or transforming Hg (Lin et al., 2012). Moreover, the enhanced familiarity with the *mer* regulatory circuit has been exploited and used to develop highly sensitive reporters of Hg in environmental samples (Selifonova et al., 1993; Virta et al., 1995). Thus, the *mer* system is the basis for the continued development of biological tools for Hg bioremediation and the management of Hg contaminated environments.

A survey of all microbial genomes performed in 2010 greatly expanded our understanding of the diversity of microorganisms that possess the *mer* detoxification machinery and identified its presence among early evolving lineages of thermophilic Bacteria and Archaea. This suggested that Hg resistance most likely originated in a hydrothermal environment, where geochemically derived Hg is at a naturally elevated concentration (Barkay et al., 2010). Unlike the *mer* operons in the *Proteobacteria*, *Firmicutes*, and *Actinobacteria*, the *mer* operons of early evolving microbial lineages encode for fewer functional genes. The regulation of *mer* functions in some of these lineages, including the *Crenarchaeota* (Schelert et al., 2006), *Aquificae* (Freedman et al., 2012), and *Thermus/Deinococcus* (Wang et al., 2009), varied from repression only (Schelert et al., 2006) to constitutive (Freedman et al., 2012). These observations led us to hypothesize that the evolution of *mer* has progressed by the sequential recruitment of functions through evolutionary time, resulting in the highly efficient and tightly regulated Hg detoxification mechanism that has been identified and examined in great detail among more recently evolved bacterial lineages (Barkay et al., 2003). Here, in an effort to better understand the evolution of Hg detoxification, we employed an integrated bioinformatic and phylogenetic approach to examine the distribution of various *mer* functions in all sequenced microbial genomes (as of December 2011) in order to reconstruct the evolutionary history of *mer* operon architecture as related to microbial evolution on Earth.

## Materials and Methods

### *mer* operon composition

A total of 272 mercuric reductase (MerA) protein homologs were compiled from all completed and publically available sequence databases using the DOE IMG and the NCBI servers in December of 2011 using tblastn and MerA from Tn*501* [CAA77323; (Stanisich et al., 1977)] and *Sulfolobus solfataricus* P2 (AAK42805; Schelert et al., 2004) as queries (Table S1 in Supplementary Material). In addition, this tabulation included several gene sequences from a number of taxa for which genomes have not been completed but for which the operon has been genetically characterized. All putative MerA sequences were examined manually for the presence of sequence signatures that have been experimentally shown to be essential for MerA activity (Barkay et al., 2003). These included the conserved cysteine pair at positions 207 and 212 [numbering in reference to MerA of *Bacillus* sp. RC607 (BAB62433)] in the redox active site, the vicinal cysteine pair at the carboxy terminus (positions 628 and 629), tyrosine at position 264 (Rennex et al., 1993), and tyrosine at position 605 for bacterial MerA and phenylalanine at position 605 for archaeal MerA (Simbahan et al., 2005). The presence of other *mer* gene homologs proximal to putative *merA* in microbial genomes was determined manually using the Neighborhood viewer on the DOE IMG server or by the Gene record function on the NCBI server. The presence of homologs of *arsR, merR* (divergent or convergent to the *merA* homolog)*, merP, merT, merC, merF, merE, merG, merH, merB, merD*, and TRASH domain-encoding genes was tabulated (Table S1 in Supplementary Material). In addition, *mer* operons were screened for multiple homologs of *merR* and *merB*, elements suggestive of horizontal gene transfer (e.g., transposition function) within ∼10 Kbp of *merA*, and were characterized as being encoded on the chromosome or on a plasmid when this information was available.

### Phylogenetic analysis of MerA and 16S rDNA

MerA protein homologs were aligned using CLUSTALX (version 2.0.8) specifying the Gonnet 250 protein substitution matrix and default gap extension and opening penalties (Larkin et al., 2007) with dihydrolipoamide dehydrogenase from *Magnetospirillum magneticum* AMB-1 (YP_423326), *Thermus thermophilus* HB27 (YP_005669), and *Pseudomonas fluorescens* Pf0-1 (YP_351398) serving as outgroups. N terminal “NmerA” sequences were trimmed from the alignment block as previously described (Barkay et al., 2010) and the phylogeny of MerA was evaluated with aBayes-PhyML (ver. 3.0.1; Anisimova et al., 2011) using the LG amino acid substitution matrix, a discrete four category gamma substitution model (gamma shape parameter = 1.078), and a defined proportion of invariant sites of 0.034, as recommended by ProtTest (version 2.4; Abascal et al., 2005). Approximate likelihood-ratio tests (aLRT) were used as an alternative to non-parametric bootstrap frequencies. A consensus phylogenetic tree was projected from 1000 aLRT permutations using FigTree (ver. 1.2.2; http://tree.bio.ed.ac.uk/UH). Trait-based analyses require a rate smoothed phylogram in order to limit biases in rapidly evolving lineages. Thus, the phylogram was rate smoothed using a penalized likelihood approach (Sanderson, 2002) as implemented by the chronopl program where a lambda smoothing parameter of 0.8 was specified over 1000 iterations. Chronopl is a part of the Ape package (ver. 3.0–3; Paradis et al., 2004) and is implemented within the base package R (ver. 2.13.1; R Development Core Team, 2010).

Representative MerP and MerT protein sequences were compiled from *mer* operons, aligned as described above, and their evolutionary history evaluated with the Neighbor-Joining method due to the high conservation in the sequences (i.e., low phylogenetic signal) and the short length of the alignment blocks (∼170 positions for each) with the program MEGA5 (Tamura et al., 2011). The consensus tree was inferred from 100 bootstrap replicates with branches with less than 50% bootstrap support collapsed. The phylogenetic distances of MerP and MerT, as computed using the Poisson correction method, is represented as the number of amino acid substitutions per site. All ambiguous positions were removed for each sequence pair.

Small subunit (SSU) rRNA genes were compiled from completed genomes that also encoded for a homolog of MerA on chromosomal DNA. SSU rRNA genes from genomes that encoded for MerA on plasmid DNA were not included in the comparison of MerA protein and SSU rRNA gene phylogenetic distances. Compiled SSU rRNA genes were aligned with CLUSTALX, specifying the IUB DNA weight matrix, and default gap opening and extension penalties with SSU rRNA genes from the eukaryotic taxa *Heteromita globosa* (U42447) and *Trissopathes pseudotristicha* isolate HAS-31 (FJ389899) serving as outgroups. The phylogeny of the SSU rRNA genes was evaluated with aBayes-PhyML (ver. 3.0.1; Anisimova et al., 2011) using the GTR amino acid substitution matrix with a discrete 4 category gamma substitution model (gamma shape parameter = 0.629) and a defined proportion of invariant sites (0.149), as recommended by jModelTest (version 0.1.1; Posada, 2008). Approximate likelihood-ratio tests (aLRT) were used as an alternative to non-parametric bootstrap frequencies. The 16S rRNA gene phylogram was rate smoothed as described above.

### Statistical analyses

A Mantel regression approach was employed to evaluate the extent to which the phylogenetic dissimilarity of MerA varied with the phylogenetic dissimilarity of SSU rRNA genes. Phylocom (ver. 4.1; Webb et al., 2008) was used to generate matrices describing Rao’s phylogenetic dissimilarity of the rate smoothed MerA protein phylogram and the SSU rRNA gene phylogram, as previously described (Boyd et al., 2011). The Mantel correlation coefficient and associated *p*-value for the relationship were determined using 1000 permutations, as implemented in the XLSTAT software package (ver. 2008.7.03). Pearson linear regressions were used to evaluate the co-occurrence of genes comprising *mer* operons. The Pearson correlation coefficient and associated *p*-value for the relationship was determined from 1000 permutations, as implemented with XLSTAT.

The individual genes that comprise the *mer* operon for each taxon were treated as binary traits, with a 1 and 0 indicating the presence and absence of the trait, respectively. Trait-based evolutionary methods were then applied to determine the extent to which the MerA rate smoothed phylogeny predicts the similarity in the distribution of the trait(s) among closely related taxa. The phylogenetic signal (*K*-statistic) for each trait was quantified using the program multiphylosignal within the Picante package (Kembel et al., 2010) as implemented with the base package R (ver. 2.13.1; R Development Core Team, 2010). The *K*-statistic compares the observed phylogenetic signal of a trait to the signal under a Brownian motion model of evolution on a phylogeny (Blomberg et al., 2003). Values of *K* that are close to one imply a Brownian motion for the evolution of a trait (or some degree of phylogenetic signal) while values greater than one indicate strong phylogenetic signal for a given trait. *K* values closer to zero or which are negative correspond to a random or convergent pattern of evolution for that trait. The statistical significance of *K* was evaluated by comparing patterns of the variance of independent contrasts of the trait on a phylogeny to a null model produced by shuffling taxa labels across the tips of the phylogeny (Kembel et al., 2010).

The complexity of *mer* operons was also evaluated using trait-based approaches. A metric describing the complexity of *mer* operons was calculated by dividing the sum of the number of *mer* homologs encoded in an operon by 13, the total number of types of *mer*-associated genes retrieved in the present study. The distribution of individual *mer*-encoded functions and the complexity of *mer* operons was mapped on the rate smoothed MerA phylogeny using the Ape package (ver. 3.0–3; Paradis et al., 2004) within the base package R.

## Results and Discussion

Here, we examine the evolution of the *mer* operon as a function of the evolutionary history of MerA. We first present the taxonomic distribution of *mer* in completed genomes and then follow this with a phylogenetic assessment of the evolution of MerA. Finally, we integrate new data describing the composition of *mer* operons into this taxonomic and phylogenetic framework and propose a paradigm for the evolution of Hg detoxification among prokaryotes.

### Taxonomic distribution of *merA*

A total of 272 MerA homologs encoding for the mercuric reductase subunit were identified in public databases as of December 2011 (Table S1 in Supplementary Material). These 272 MerA protein homologs were distributed among 246 genomes, with 23 of these genomes encoding for multiple (two or three) homologs. MerA protein homologs were identified in both archaeal as well as bacterial genomes, but were not identified in eukaryal genomes. Among the Archaea, homologs of MerA were identified among the *Crenarchaeota* (15/21 available genomes) and the *Euryarcheota* (8/34 available genomes). Among the Bacteria, homologs of MerA were prevalent among members of the *Aquificae* (3/7 genomes), *Actinobacteria* (29/91 genomes), *Firmicutes* (42/328 genomes), and *Proteobacteria* (140/517 genomes). In addition, homologs of MerA were identified in the genomes of members of the *Bacteroidetes*, *Chloroflexi*, *Deinococcus*/*Thermus*, *Tenericutes*, *Nitrospira*, and *Verrucomicrobia*. As we have noted before, MerA was not found in entire microbial taxa and guilds, most notably among phototrophs and *Epsilon-proteobacteria*, and were only rarely identified among obligate anaerobes (Barkay et al., 2010).

### MerA phylogeny

**Figure 2:**
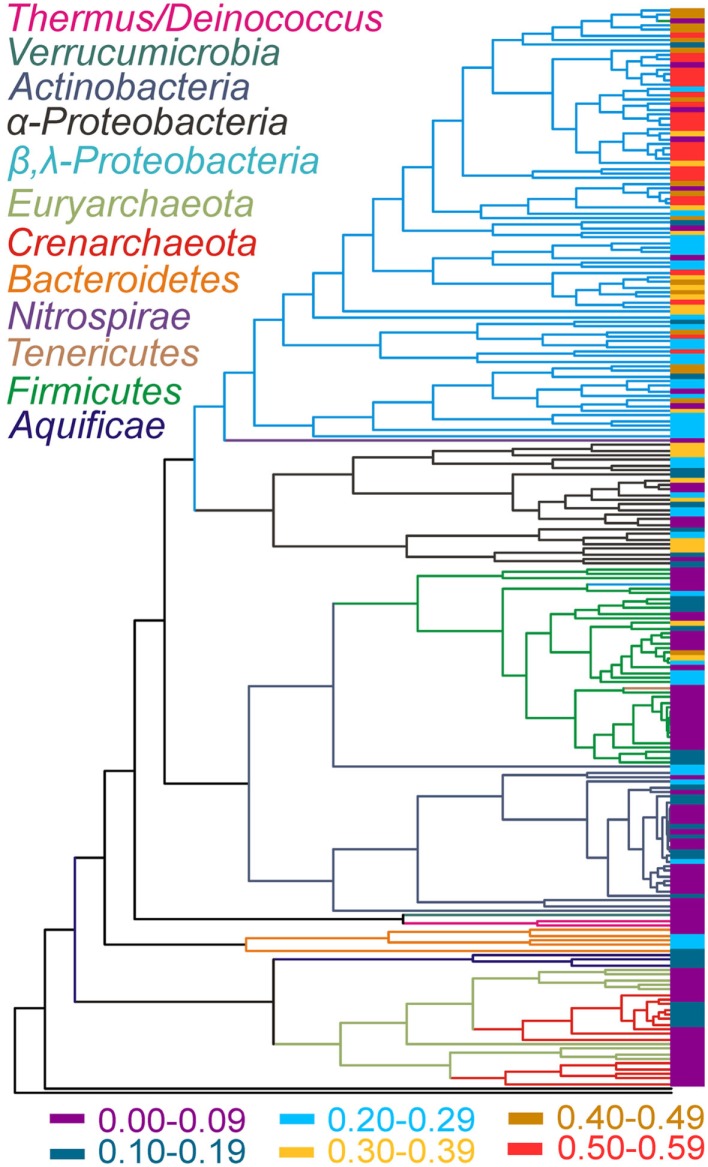
**Rate smoothed MerA phylogenetic tree (plasmid MerA were not included) with the complexity index of the *mer* operon associated with each MerA terminal indicated**. The complexity index (indicated by the color at the sequence terminals and defined at the bottom) was calculated as the sum of the presence (value of 1) or absence (value of 0) of individual *mer* genes divided by 13 (a total of 13 individual *mer*-encoded genes). Lineages are color coded to indicate the phylum level taxonomy of each lineage with the exception of the proteobacterial phyla, which was further delineated to indicate *Alpha* (α)- and *Beta*/*Gamma* (β/λ)-*proteobacteria*.

The phylogenetic relationships of chromosomally encoded MerA proteins were determined for use in examining patterns in the evolution of the *mer* operon (Figure 2). Plasmid encoded MerA proteins were excluded from this analysis, an exclusion that should have little effect on overall conclusions (see below and Figure S1 in Supplementary Material), in order to facilitate a clear demarcation of the MerA phylogeny as it relates to species (16S rRNA gene) phylogeny. Consistent with prior analyses (Barkay et al., 2010; Freedman et al., 2012), phylogenetic reconstruction of chromosomally encoded MerA reveals well supported lineages that generally correspond with the phylum level taxonomic rank of the genomes from which they originate. The updated phylogeny consists of two large clusters, one consisting of all bacterial sequences, with the exception of *Aquificae* MerA. *Aquificae* MerA form a basal branching sub-lineage in a second large cluster that is primarily comprised of archaeal homologs. Thus, bacterial MerA is paraphyletic with respect to archaeal MerA, with *Aquificae* MerA branching basal to archaeal MerA indicating a bacterial origin for this enzyme. The archaeal lineage is paraphyletic with respect to the *Crenarchaeota* and the *Euryarchaeota*, with two distinct crenarchaeal and three distinct euryarchaeal lineages. Two of the euryarchaeal lineages nest the two crenarchaeal lineage. Together, the tree topology (Figure 2) may suggest that *merA* was first acquired within the *Euryarchaeota* by a lateral gene transfer (LGT) event with an ancestor of the *Aquificae* followed by multiple transfers between the *Euryarchaeota* and *Crenarchaeota*. Additional examples of LGT events are evident in the evolution of chromosomally encoded MerA within the more recently evolved bacteria, in particular between the *Beta*- and the *Gamma-proteobacteria*. Other examples include the cross-phylum LGT in the tenericute *Acholeplasma laidlawii* PG-8A that likely obtained MerA from a member of the *Firmicutes*, and the early transfer event to *Nitrospirae* from a member of the *Proteobacteria* (Figure 2). Thus, while the overall MerA phylogeny corresponds to the phylum level species phylogeny, several examples of LGT events are evident. In addition, an examination of the genomic environment flanking *mer* operons (only complete genomes examined) reveals evidence of recombination events as indicated by a number of examples of the presence of genes encoding transposases, resolvases, inverted repeats, and phage genes (Table S1 in Supplementary Material).

**Figure 3:**
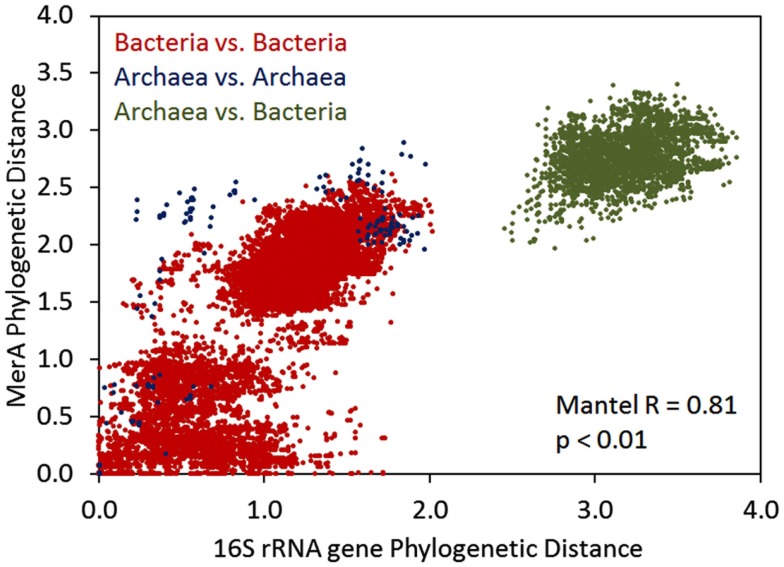
**Mantel regression of a matrix describing Rao’s phylogenetic distance among MerA proteins and a matrix describing Rao’s phylogenetic distance among 16S rRNA genes in all chromosomally encoded *merA***. Color overlays indicate the pairwise comparisons that are being made.

We further examined the relationship between the evolution of MerA and the evolution of the organisms whose genomes encode for this function by pairwise comparisons of evolutionary distances among MerA and SSU rRNA genes, in taxa whose genomes encode for chromosomal MerA (Figure 3). A Mantel regression approach was utilized in order to identify phylogenetic barriers to gene flow among domains (Archaea and Bacteria) of organisms that encode for MerA. Here, pairwise comparisons that reveal closely related MerA genes (low phylogenetic distance) and divergent SSU rRNA genes (high phylogenetic distance) would be indicative of LGT. Broadly, the results of the Mantel test reveal that the evolution of chromosomal MerA and the evolution of the 16S rRNA gene of taxa is significantly correlated (Mantel *R* = 0.81, *p *< 0.01), suggesting an overarching role for vertical inheritance in the evolution of MerA, at least in chromosomally encoded MerA. When only bacterial MerA/16S rRNA genes are considered, the correlation is stronger (Mantel *R* = 0.79, *p *< 0.01) than when only archaeal MerA/16S rRNA genes are considered (Mantel *R* = 0.65, *p *< 0.01), a difference that could be attributed to the large evolutionary distances associated with MerA derived from the genomes of closely related *Crenarchaea* as depicted by several points representing short 16S rRNA gene distances with long MerA distances (Figure 3). The frequent LGT between the Archaeal phyla (see above) may account for this observation. The occurrence of LGT of *merA* among the Bacteria is also evident in the bacterial 16S rDNA vs. MerA pairwise plot with a number of points representing small MerA phylogenetic distances [<0.1 relative evolutionary units (U)] and large 16S rDNA phylogenetic distance (>1.5 U), supporting previous evidence indicating that MerA in these taxa were derived by recent LGT events (Barkay et al., 2010; Lal and Lal, 2010).

### Gene complement of *mer* operons

Genes flanking MerA homologs were examined for their homology to ancillary functions involved in the regulation of *mer* (*arsR*, *merR, merD*), Hg binding and/or transport (*merT*, *merP*, *merC*, *merF*, *merE*), metal sensing (TRASH), and organomercury detoxification functions (*merB, merG*). The regulatory genes *arsR*, *merR*, and *merD* were present in 22 (8.1% of total), 195 (71.7%), and 78 (28.7%) *mer* operons (Table 1) and with the exception of only two taxa (*Bacillus cellulosilyticus* DSM 2522 and *Gordonia bronchialis* DSM 43247), each operon encoded for either *merR* or *arsR* (Table S1 in Supplementary Material). *merR* was detected in a convergent transcriptional orientation (52 total occurrences) and more commonly in a divergent orientation (143 total occurrences), with respect to the orientation of the rest of the *mer* gene cluster. Genes encoding proteins implicated in Hg binding and/or transport [*merT*, *merP*, *merC*, *merF*, and *merE* (Hamlett et al., 1992)], were present in 147 (54.0% of total), 134 (49.3%), 56 (20.6%), 25 (9.2%), and 63 (23.2%) of the *mer* operons examined, respectively. Genes encoding proteins with stand-alone TRASH domains, previously proposed to function in Hg trafficking in *mer* operons of *Sulfolobus* spp. (Ettema et al., 2003; Schelert et al., 2006) were detected in five archaeal *mer* operons (1.8% of total operons examined), but were never detected in bacterial *mer* operons. Broad spectrum Hg resistance protein-encoding genes *merB* and *merG* were less frequently detected than genes encoding for narrow spectrum Hg resistance (e.g., *merA*), as indicated by the identification of these genes in only 42 (15.4% of total), and 5 (1.8%) of the *mer* operons examined, respectively (Table S1 in Supplementary Material).

**Table 1:** **Phylogenetic signal (*K* value) and associated *p*-value of *mer* operon functions when mapped on a MerA phylologenetic tree**.

Locus and/or trait	Plasmids included					Plasmids not included				
	*K*	*p*-value	Genomic distribution			*K*	*p*-value	Genomic distribution		
			Presence	Absence	% of Total			Presence	Absence	% of Total
*arsR*	0.016	0.255	22	250	8.1	0.024	0.505	21	198	9.6
*merR*-convergent	0.014	0.082	52	220	19.1	0.034	0.157	48	171	21.9
*merR*-divergent	0.019	0.004	143	129	52.6	0.061	0.005	105	114	47.9
*merR* (multiple copies)	0.060	0.305	5	267	1.8	0.110	0.315	3	216	1.4
*merR* (both orientations)	0.012	0.152	195	77	71.7	0.034	0.353	153	66	69.9
*merP*	0.026	0.003	134	138	49.3	0.237	0.001	98	121	44.7
*merT*	0.012	0.022	147	125	54.0	0.017	0.515	109	110	49.8
*merC*	0.033	0.015	56	216	20.6	0.302	0.001	38	181	17.4
*merF*	0.108	0.002	25	247	9.2	0.307	0.001	17	202	7.8
*merE*	0.009	0.192	63	209	23.2	0.132	0.002	38	181	17.4
*merG*	0.093	0.171	5	267	1.8	0.087	0.488	2	217	0.9
*merH*	0.129	0.427	1	271	0.4	NA	NA	0	219	0.0
*merB*	0.066	0.004	42	230	15.4	0.076	0.032	33	186	15.1
*merB* (multiple copies)	0.126	0.088	5	267	1.8	0.116	0.229	4	215	1.8
*merD*	0.098	0.001	78	194	28.7	0.289	0.001	45	174	20.5
TRASH	0.141	0.054	5	267	1.8	0.141	0.139	5	214	2.3

*The distribution of *mer* functions (Presence/Absence) and % of total *mer* operons encoding for a given function are indicated. Calculations were performed with all available *mer* operons (e.g., plasmids included) as well as with *mer* operons that were only encoded on chromosomes only (e.g., without plasmids)*.

Of the 272 *mer* operons identified, 53 were located on plasmids. To determine if the presence of individual *mer* proteins in each operon depended on whether they were plasmid or chromosomally encoded, we regressed the relative abundance of each individual *mer*-encoded protein in the “Plasmid Included” and “Plasmid Not Included” databases (Figure S1 in Supplementary Material). If there is no bias in the distribution of *mer* functions on plasmid or chromosomal DNA, then the expected slope of the regression should be 1.0 (1:1 relationship). Deviations from this 1:1 relationship would be indicative of genes that tend to be encoded on plasmid DNA or chromosomal DNA, depending on which side of the regression line they plotted. The slope of the regression analysis depicted [1.05 (Figure S1 in Supplementary Material)], was slightly higher than the expected value of the unbiased 1:1 ratio, suggestive of several proteins being more commonly encoded on plasmids. The Hg binding and/or transport functions *merE*, *merC*, *merP*, and *merT* as well as *merD*, a regulatory protein, all plot slightly above the 1:1 line indicating that they are more likely to be plasmid encoded. Indeed, the presence of all of these co-varied in a significant and positive relationship with their derivation from plasmid DNA (Pearson *R* = 0.372–0.169, *p *< 0.005; Table S2 in Supplementary Material). In contrast, *arsR* and convergent *merR* (convergent orientation relative to the direction of other proximal *mer* genes) plot below the 1:1 line, which suggests that these proteins tend to be encoded on chromosomal DNA. Thus, the analysis suggests that several *mer*-encoded functions (e.g., *merE*, *merC*, *merP*, and *merT* as well as *merD*) are more likely than others (*arsR* and *merR*) to be encoded on plasmids. Such observations are likely to have important ramifications for the acquisition of these genes via LGT, as discussed in more detail below. Importantly, the deviations from the 1:1 line noted above are not large, suggesting that the composition of plasmid and chromosomal *mer* operons are broadly similar. This observation justifies the use of either database, plasmid genes included or excluded, for further trait-based analyses as presented below.

### Evolution of the *mer* Operon

**Figure 4:**
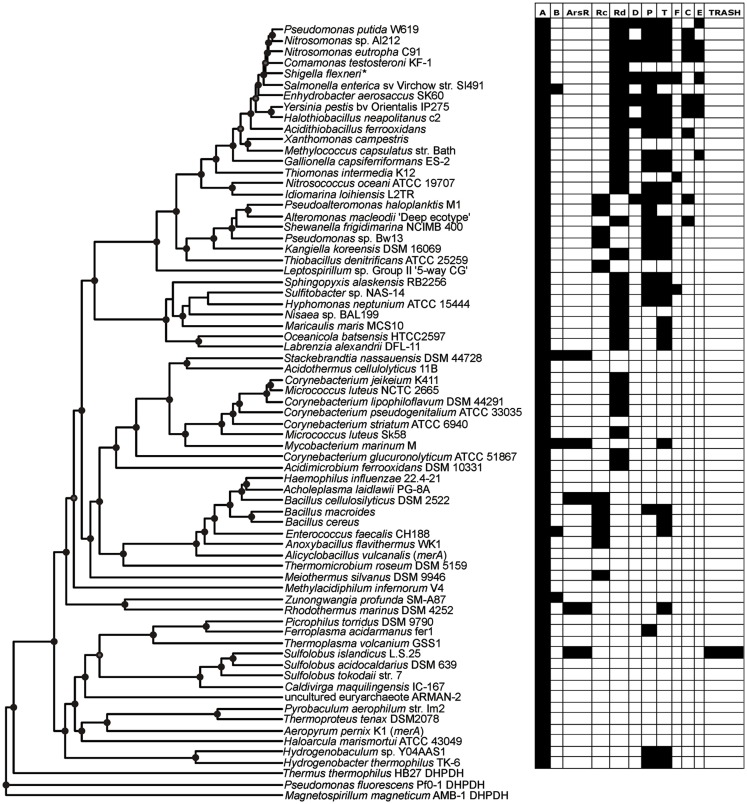
**Rate smoothed phylogenetic tree of representative MerA and the distribution of individual *mer* functions encoded in the associated operon**.

Patterns in the composition of the *mer* operon as a function of the evolutionary history of MerA were initially examined by mapping a metric that describes its complexity (e.g., total number of *mer*-encoded genes) on the MerA phylogenetic tree (side bar in Figure 2). Qualitatively, this analysis indicates that the complexity of the *mer* operon has grown as MerA evolved, with early evolving lineages (e.g., *Aquificae*, *Crenarchaeota*, *Euryarchaeota*) tending to harbor less complex *mer* operons than more recently evolved lineages (e.g., *Proteobacteria*). In order to identify which genes are contributing to the increased complexity as well as to define the composition of the ancestral *mer* operon, the distribution of individual *mer*-encoded proteins was mapped on the chromosomal MerA phylogenetic tree (Figure 4, Figures S2–S13 in Supplementary Material) and the co-variation in their distribution evaluated using linear regression approaches (Table 2).

**Table 2:** **Pearson correlation coefficients (*R*) and associated *p*-values indicating the co-variation in the presence/absence of individual *mer* operons, as assessed using linear regression**.

	*merR*(conv)	*merR*(div)	*merR*(all)	*arsR*	*merP*	*merT*	*merC*	*merF*	*merE*	*merG*	*merB*	*merD*	TRASH
**PEARSON *R***													
*merR*(conv)	1.00	−0.51	0.35	−0.14	−0.03	−0.06	−0.13	−0.07	−0.01	0.07	−0.07	−0.13	−0.08
*merR*(div)		1.00	0.63	−0.25	0.44	0.45	0.26	0.23	0.26	0.00	0.13	0.35	−0.15
*merR*(all)			1.00	−0.39	0.45	0.43	0.17	0.19	0.27	0.06	0.08	0.26	−0.23
*arsR*				1.00	−0.29	−0.17	−0.15	−0.09	−0.15	−0.03	0.17	−0.17	0.47
*merP*					1.00	0.78	0.36	0.19	0.39	0.11	0.06	0.47	−0.14
*merT*						1.00	0.32	0.09	0.32	0.10	0.14	0.44	−0.15
*merC*							1.00	−0.13	0.40	−0.04	−0.06	0.60	−0.07
*merF*								1.00	0.00	−0.03	−0.03	0.02	−0.04
*merE*									1.00	0.08	0.04	0.63	−0.07
*merG*										1.00	0.23	0.19	−0.01
*merB*											1.00	0.07	−0.06
*merD*												1.00	−0.08
TRASH													1.00
***p*-VALUES**													
*merR*(conv)	<0.01	<0.01	<0.01	0.05	0.63	0.35	0.06	0.29	0.89	0.34	0.31	0.05	0.23
*merR*(div)		<0.01	<0.01	0.00	<0.01	<0.01	<0.01	<0.01	<0.01	0.95	0.05	<0.01	0.03
*merR*(all)			<0.01	<0.01	<0.01	<0.01	0.01	0.00	<0.01	0.35	0.23	<0.01	0.00
*arsR*				<0.01	<0.01	0.01	0.03	0.16	0.03	0.65	0.01	0.01	<0.01
*merP*					<0.01	<0.01	<0.01	0.01	<0.01	0.12	0.40	<0.01	0.04
*merT*						<0.01	<0.01	0.20	<0.01	0.15	0.04	<0.01	0.02
*merC*							<0.01	0.05	<0.01	0.52	0.39	<0.01	0.30
*merF*								<0.01	0.97	0.68	0.69	0.75	0.51
*merE*									<0.01	0.22	0.53	<0.01	0.30
*merG*										<0.01	0.00	0.01	0.83
*merB*											<0.01	0.30	0.34
*merD*												<0.01	0.25
TRASH													<0.01

*Only mer operons that were encoded on a chromosome were considered. An analogous analysis with all *mer* operons included is presented in Table S2 in Supplementary Material*.

The earliest evolving bacterial *mer* operons, those associated with the *Aquificales*, are comprised of *merA* and the metal binding protein-encoding genes, *merP* (periplasmic Hg scavenging function) and *merT* (Figure 4). Intriguingly, the *mer* operons associated with the Archaea, which clearly acquired *mer* from an ancestor of the *Aquificae* (See above), are simpler than those of the *Aquificae*. The Archaeal *mer* operons are often comprised of only *merA*, and in the case of several *Sulfolobus* strains, an *arsR* regulator and a TRASH metal sensing domain protein (Schelert et al., 2006). TRASH was not found in association with any *mer* operons with the exception of *Sulfolobus* spp. and *arsR* was present in six of eight representative of this genus (Table S1 in Supplementary Material) leading to strong positive correlation between the two (Pearson *R* = 0.47, *p *< 0.01) and strongly negative correlations between TRASH and *merR*, *merT*, and *merP* (Pearson *R* = −0.23 to −0.14, *p *= 0.01–0.03).

With the exception of a single euryarchaeal taxon (*Ferroplasma acidarmanus* fer1) that encodes for a *merP* homolog, these transporters are absent from all taxa within the Archaea. While the absence of membrane- and periplasm-associated *mer* functions is expected based on the difference in cell wall structures between the domains, this finding suggests that *merP* and *merT* (i) may not have been transferred during the LGT event that gave rise to *merA* in the Archaea, (ii) were purged from the genome of an ancestral Archaeon resulting in its near universal absence among extant sequenced genomes, or (iii) were acquired in the *Aquificales* via LGT. In order to better establish which of these possibilities is most likely, we reconstructed the evolutionary histories of MerP and MerT. Phylogenetic reconstruction of MerP (Figure S14 in Supplementary Material) and MerT (Figure S15 in Supplementary Material) revealed that those proteins derived from the *Aquificales* are monophyletic and branched basal to all other bacterial proteins even though many instances of LGT are clearly evident in the MerP and MerT phylogenies. This observation rules out scenario (iii) above and instead suggests a vertical line of inheritance within the *Aquificae* and a recruitment to the *mer* operon early during its evolution. Importantly, MerP from *F*. *acidarmanus* fer1 is nested among *Aquificae* MerP which indicates that MerP in this taxon was likely obtained through LGT; possibly the same event that led to the transfer of *merA*. Thus, available data suggests that the most plausible scenario is (ii) whereby *merP* and *merT* were lost following the LGT event that gave rise to *mer* among the Archaea. This scenario is further bolstered by the previously documented central role of gene loss in the evolution of microbial genomes (Ochman et al., 2000; Morris et al., 2012). Moreover, this finding adds further support to the hypothesis that *mer* evolved among the *Aquificae* and was laterally transferred to the Archaea via interaction with an ancestral euryarchaeon.

The increased complexity of *mer* operons with the evolution of MerA can be attributed to the gradual addition of functions involved in the regulation of the operon by Hg, Hg transport, and organomercury resistance (Figures 2 and 4). *mer* operons associated with the *Aquificales* lack homologs of regulatory elements (Figure 4, Figure S2 and Table S1 in Supplementary Material) and recent experimental evidence suggests that exposure to sub lethal concentrations of Hg did not induce further expression of *merA* in *Hydrogenivirga* sp. 128-5-R1-1 and *Hydrogenobaculum* sp. Y04AAS1 (Freedman et al., 2012), suggesting constitutive expression. In contrast, *mer* operons associated with a number of *Euryarchaeota* and *Crenarchaeota* encode for ArsR-like regulators as do *mer* operons associated with the *Bacteroidetes* [which branch basal to all bacterial MerA lineages in the large bacterial clade of the tree (Figure 2)], a number of *Actinobacteria*, as well as a few firmicutes (Table S1 in Supplementary Material). ArsR is a regulator that, in the absence of Hg, represses *mer* expression in *S. solfataricus* P2 (Schelert et al., 2006) and in *Streptomyces lividans* (Brunker et al., 1996). ArsR is completely absent in all *mer* operons associated with other phyla, most notably the large *Proteobacteria* clades. In the *Proteobacteria*, as well as among most of the *Firmicutes*, ArsR-like regulatory elements are replaced by MerR (Figures S2, S10, and S11 in Supplementary Material). MerR represses *mer* operon transcription in the absence of Hg and induces expression in its presence (Summers, 1992; Guo et al., 2010), enhancing gene expression by several orders of magnitude in response to Hg exposure (Barkay et al., 2003). Finally, a second regulatory function, MerD, implicated in the down regulation of *mer* expression following decline in intracellular Hg pools (Mukhopadhyay et al., 1991) is only present in the *Beta*- and *Gamma-proteobacteria*, indicating a relatively recent origin for this function. As a result of the clear distribution patterns of regulatory elements among taxa, strong inverse correlations were observed (Table 2) between the presence/absence of *arsR* and *merR* [both orientations (Pearson *R* = −0.39, *p *< 0.01)] and *arsR* and *merD* (Pearson *R* = −0.17, *p* = 0.01) in *mer* operons.

Another distinction of *mer* operon regulation is the divergent vs. convergent orientation of *merR* relative to that of other *mer* genes. The earliest evolving MerA proteins that also encode for MerR regulatory elements are found in the *Thermus*/*Deinococcus* lineage and are transcribed convergently with *merA* (Wang et al., 2009). Convergently transcribed regulatory elements predominate among the *Firmicutes*, are rare in the *Beta*- and *Gamma-proteobacteria*, and are missing among the *Alpha-proteobacteria* and the *Actinobacteria* (Table S1 in Supplementary Material). Divergently transcribed regulatory elements emerged shortly after convergent *merR* in the *Actinobacteria*, and are common in the *Proteobacteria*. Despite the non-conformity in the inheritance of the orientation by which *merR* is transcribed, that the variation is rooted relatively deep in the evolutionary history of *mer* is indicated by the phylum level coherence in transcription orientation. The biological meaning of convergent vs. divergent evolution is the opportunity afforded by the later to differentially express *merR* and functional proteins of the operon. Thus, in proteobacterial operons MerR acts as a repressor of its own transcription regardless of the presence of Hg while it is a repressor-activator of the rest of the divergently transcribed genes (Summers, 1992). A positive correlation (Table 2) between *merD* and *merR*-divergent (Pearson *R* = 0.35, *p *< 0.01) and an inverse correlation between *merD* and *merR*-convergent (Pearson *R* = −0.13, *p *= 0.05) reflect the later diversification of both *merD* and *merR*-divergent relative to *merR*-convergent. Together, it seems that the evolution of the regulation of *mer* operon expression has progressed from constitutive expression in the *Aquificales*, organisms whose natural habitats include sulfidic and often low pH springs (Huber and Eder, 2006) where geologically derived Hg concentrations are elevated and likely constantly present (King et al., 2006; Boyd et al., 2009), to repressed expression of operons associated with early lineages of archaeal and bacterial MerA, to an efficiently regulated system among aerobes that may be intermittently exposed to high concentrations of Hg in contaminated environments. This emerging paradigm optimizes the balance between the high costs of maintaining and expressing *mer* operon proteins and the high toxicity of Hg to microbiota.

A large number of proteins that are involved in metal binding and/or transport exhibit distributional patterns on the MerA tree that corroborate an overall increased complexity of the *mer* operon as a function of MerA evolutionary time. Genes encoding for the periplasmic Hg scavenging protein, MerP, and the inner membrane spanning protein, MerT, are by far the most common (Table 1; Table S1 in Supplementary Material), present in the earliest *Aquificae* operons, and are distributed throughout the tree (Figure 4). In contrast, genes encoding alternative transporters, MerC, MerF, MerE, and MerH, are qualitatively more prevalent in operons associated with more recently evolved MerA lineages. For example, *merC* (Figure S4 in Supplementary Material) is only present in *mer* operons associated with *Beta*- and *Gamma-proteobacteria*. Both *merE and merF* homologs exhibit a patchy distribution among the *Firmicutes* and *Proteobacteria* (Figures S6 and S7 in Supplementary Material), but are absent in early evolving taxa. These transporters vary in the number of predicted membrane spanning domains [three in MerT, four in both MerC and MerH, and two in both MerF and MerE (Barkay et al., 2003; Lin et al., 2012)] and in substrate specificity, with MerE specific to inorganic and MeHg and MerT to inorganic and arylmercury (e.g., phenylmercury acetate), but not to MeHg [summarized in (Lin et al., 2012)]. Co-varying patterns of distribution of Hg transporters/binding functions and other *mer-*encoded functions (Table 2), reflect both known interactions [e.g., the positive correlation between MerP and MerT (Pearson *R* = 0.78, *p *< 0.001)] and with the relative time of their appearance during the evolution of MerA, as exhibited by strong and inverse correlations between ArsR and all transport/binding functions (Pearson *R* = −0.29 to −0.14, *p *< 0.01–0.03).

The distribution of genes that encode proteins involved in broad spectrum Hg resistance, and which are related to organomercurial degradation (MerB, MerG), also exhibited a patchy distribution with respect to the evolution of MerA. *merB* genes were identified among members of the *Bacteroidetes*, *Actinobacteria*, *Firmicutes*, and *Proteobacteria*. The *merG* gene, which encodes a protein thought to be involved in reducing cellular permeability of phenylmercury (Kiyono and Pan-Hou, 1999), was only identified in five operons, three of which originated in pseudomonads with the remaining two detected on plasmids that were directly isolated from soil (Table S1 in Supplementary Material). *merG* gene homologs were never identified in *mer* operons that did not also encode for *merB* genes (hence the strong positive correlation between the two, Pearson *R* = 0.23, *p *= 0.01), consistent with their documented function in attenuating the toxicity of organomercurial compounds (Kiyono and Pan-Hou, 1999). Importantly, the recruitment of MerG protein-encoding genes to the *mer* operon and the enhanced protection that this gives cells to the toxic effects of organomercurials, coupled with the fact that *merG* genes are only present in more recently evolved taxa, suggests this to be another example of how the *mer* detoxification system has been refined through evolutionary time and possibly in response to the release of anthropogenic organomercury contaminants.

### The role of plasmids in the evolution of the *mer* operon

The qualitative patterns observed in the distribution of *mer* functions in chromosomally encoded genes, as outlined above, were quantified using trait-based modeling tools. Here, each gene was treated as a “trait” and the dispersion of traits was mapped on the rate smoothed MerA phylogram and were then quantified. The *K*-statistic compares the observed signal of a trait to the signal under a Brownian motion model of evolution on a phylogeny (Blomberg et al., 2003). Values of *K* that are close to one imply a Brownian motion for the evolution of a trait (or some degree of phylogenetic signal) while values greater than one indicate strong phylogenetic signal for a given trait. *K* values closer to zero or which are negative correspond to a random or convergent pattern of evolution for that trait. Variation in the *K*-statistic when only chromosomal *mer* operons were considered as compared to values obtained when all *mer* operons (chromosomal and plasmid-origin) were considered, are attributable to plasmids obscuring vertical patterns of inheritance. In such a scenario, decreases in *K* are indicative of plasmid-based LGT events decreasing the signal for vertical inheritance.

**Figure 5:**
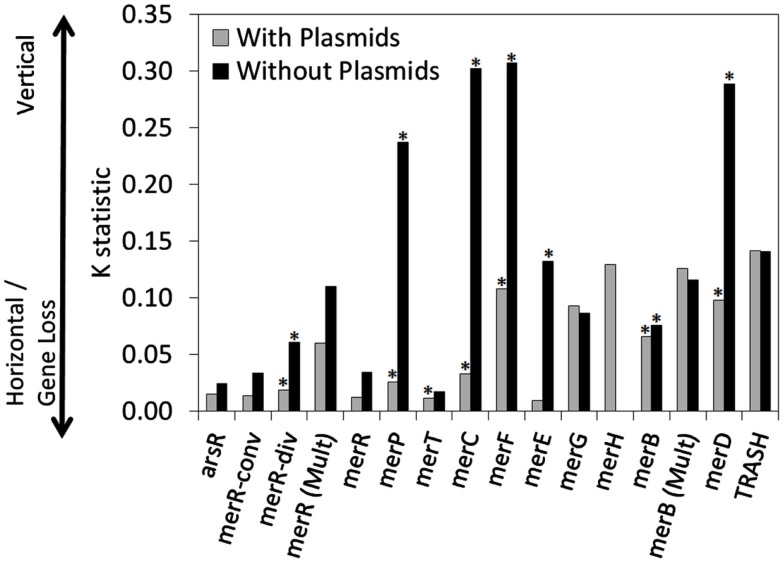
**The *K*-statistic associated with individual *mer* functions, when mapped on the rate smoothed MerA phylogenetic tree**. *K*-statistics were calculated for *mer* functions encoded on operons that were either chromosomal encoded (e.g., without plasmids) or that were chromosomal and plasmid encoded (with plasmids). *K* values that were statistically significant (*p*-value < 0.05) are indicated with an asterisk. Low *K* values are suggestive of a strong tendency for the gene to be subjected to LGT or gene loss. In contrast, high *K* values are suggestive of vertical transmission of the gene.

A number of chromosomal *mer*-encoded genes exhibited statistically significant and positive *K* values, including *merP* (*K* = 0.237, *p* < 0.001), *merC* (*K* = 0.302, *p* < 0.001), *merF* (*K* = 0.307, *p* < 0.001) and *merD* (*K* = 0.289, *p* < 0.001; Table 1; Figure 5), suggesting a tendency for these genes to be transferred vertically through chromosome-based replication and diversification. However, when the phylogenetic distribution of plasmid-based *mer* functions was also included in the analysis, the *K*-statistic decreased dramatically for these functions (*K* = 0.026, 0.033, 0.108, 0.098, respectively). This large decrease in the *K*-statistic indicates that the pattern of vertical inheritance of these genes was obscured by the inclusion of plasmid-based sequences, indicating a prevalent role for plasmids in the dispersion of *mer* operons and individual *mer* functions across phylogenetic boundaries. Indeed, a large decrease in the *K*-statistic was also observed in the case of *merR* (conv), *merR* (div), *merR* (total), *merT*, *merE*, and *merB*, with several of these decreases being statistically significant [*merR* (div), *merT*, *merE*, and *merB*]. Due to the fact that the *K*-statistic is sensitive to the phylogenetic distance between and among organisms that share or lack a given trait (i.e., *mer*-encoded gene; Blomberg et al., 2003), those genes with *K* values that underwent the most significant decreases when plasmid-based *mer* were included in the analysis (Figure 5) can be considered to be those which are least sensitive to phylogenetic boundaries in their dispersion. The *mer* genes that underwent the largest decrease in the *K*-statistic were the genes encoding for proteins involved in Hg transport/binding *merC*, *merP*, *merF*, and *merE*, and regulation, *merD*. Qualitative examinations of the distribution of these genes on the rate smoothed MerA phylogeny (Figures S4–S7 and S9 in Supplementary Material, respectively) reveal a limited and patchy distribution of these genes within one or two closely related phyla. Such observations, coupled with the results of the differential *K*-statistic when plasmids are included/excluded, suggests that plasmid-based LGT is a likely mechanism influencing the evolution of the *mer* operon.

None of the *K* values obtained in the present analyses of individual *mer* operon functions (both plasmid encoded *mer* operons included and excluded), when mapped on the MerA phylogram, yield particularly high *K* values that would be indicative of an overall pattern of vertical inheritance, with minimal influence from LGT. This is in stark contrast to previous analyses of the evolution of nitrogenase, which converts dinitrogen gas to ammonia (Rubio and Ludden, 2008), as well as Suf proteins, which are involved in iron sulfide cluster biosynthesis (Outten et al., 2004), where strong evidence (*K* values > 1.0) for vertical descent in ancillary and core functions has been previously observed (Eric S. Boyd, *unpublished data*). This observation, suggest that although the evolution of MerA, the core of the mercury detoxification mechanism, is likely to have occurred primarily by vertical descent, a number of the ancillary functions have been subjected to extensive LGT or gene loss. This is likely due to the high energetic costs of maintaining ancillary functions that are not required for enzymatic activity, resulting in a strong selective pressure to purge genomes of ancillary functions that lead to an overall decrease in organismal fitness (Morris et al., 2012).

### The role of LGT in *mer* operon evolution

In contrast to ancillary *mer*-encoded functions, which appear to be subject to extensive gene loss and transfer events, evidence for an extenstive role of LGT in the evolution of MerA is relatively scarce. The relative scarcity for LGT of MerA, which is subject to a strong positive selection (Gogarten et al., 2001), is an enigma which we previously attributed to the co-varying effects of environmental conditions on Hg bioavailability and microbial species distribution (e.g., tendency for phylogenetically related taxa to inhabit similar ecological niches; Barkay et al., 2010). In addition to selective pressure, several other processes affect the extent of LGT [reviewed by Thomas and Nielsen (2005)] and the integration and stable inheritance of the transferred genetic material in the recipient genome [reviewed by Boto (2009)], the consequences of which are detected by phylogenetic reconstructions (Ragan, 2001). The phylogenetic distance between taxa has been proposed as a modulator of inheritance by LGT so that while rare events of cross-domain transfer are known, the frequency of LGT events increases as the phylogenetic distance between donor and recipient declines (Gogarten et al., 2001; Boto, 2009). When tested, the relative frequency of within phylum as compared to cross-phylum LGT has been shown to be dependent on the phyla considered (Zhaxybayeva et al., 2006, 2009). Careful examination of the *Alpha-proteobacteria* MerA clade, for which our current and previous analyses indicate an almost full congruence between the gene and the species trees [Figures 2 and 4; (Freedman et al., 2012) Figure S1 in Supplementary Material; (Barkay et al., 2010) Figure 1)], however, did reveal several cross-order transfers among marine, and between soil and marine, strains. The other congruent cluster, the *Actinobacteria*, consisted in its entirety of the order *Actinomycetales*. We therefore conclude that while LGT seems a forceful process leading to horizontal spread of *mer* genes as evident by frequent plasmid carriage (Figure 5) and the genomic proximity to signatures of past LGT events [e.g., transposases, resolvases (Table S1 in Supplementary Material)], the evolutionary signals of such events on the evolution of MerA have ameliorated due to post transfer selection exerted by the environment as well as by the intracellular constraints on *mer* functions, e.g., MerA dependence on intracellular redox buffering (Ledwidge et al., 2005) and interactions with membrane constituents of Hg transporters (Barkay et al., 2003).

## Conclusions and Relevance to Mercury Bioremediation

The Hg resistance system, as present in a large number of extant microorganisms, is a modular system (Liebert et al., 2000) that has been assembled over evolutionary time spans by strong selection and enhanced fitness in environments with varying patterns of exposure to Hg. Initially limited to geothermal environments where exposure to geological sources of Hg has driven the derivation of MerA likely through ancient gene duplication and subsequent mutations of another flavin-nucleotide disulfide oxidoreductase (Pullikuth and Gill, 1997), *mer* operons have gradually increased their gene complements and functional diversity (Figures 2 and 4), in particular with respect to the efficiency of regulatory control on gene expression. A simple constitutively expressed system affording resistance in environments with constant exposure to Hg has become a tightly regulated efficient Hg detoxification machine in diverse environments.

The database studied here was established based on annotation of ORF’s proximal to *merA* as *mer* gene homologs and it is therefore limited to functions previously identified to play a role in Hg detoxification. A limitation of this approach is the potential omission of genes, and the functions they specify, that are a part of uncharacterized *mer* operons. During our genome surveys we noticed the presence of many gene homologs, some related to metal sensing and trafficking or to cysteine biosynthesis, embedded within putative *mer* operons [for examples see Table S1 in Supplementary Material in Barkay et al. (2010)]. These homologs, especially those shown to be co-transcribed with *merA* in response to Hg(II) exposure (Schelert et al., 2006; Wang et al., 2009), may play various uncharacterized roles in the response to Hg toxicity. It is likely that once characterized, these traits will expand our understanding of the *mer* paradigm and its evolution.

With the exception of the widespread oxygenation of the Earth’s biosphere which increased the bioavailability of Hg in its most oxidized form (Barkay et al., 2010), it is currently not possible to relate events in *mer* operon evolution to an absolute time frame and/or specific events in Earth history. It is, however, plausible that industrial activities resulting in the release of Hg to natural waters and soils have accelerated the evolution of *mer* systems due to ever-increasing selective pressure associate with the use of mercurial compounds as catalysts, disinfectants, pesticides, and herbicides (Liu et al., 2012). The emerging efficient detoxification machine that converts highly toxic Hg(II) and organomercury compounds to less reactive and volatile Hg(0) has provided important tools in bioremediation and the environmental management of Hg contamination. These applications are only possible thanks to the detailed understanding of how *mer* systems function and evolve in response to the ever-increasing challenge of Hg to life.

## Conflict of Interest Statement

The authors declare that the research was conducted in the absence of any commercial or financial relationships that could be construed as a potential conflict of interest.

## Supplementary Materials

The Supplementary Material for this article can be found online at http://www.frontiersin.org/Microbiotechnology,_Ecotoxicology_and_Bioremediation/10.3389/fmicb.2012.00349/abstract

Supplementary **Table S1** ***mer* operons included in this study**.

Supplementary **Table S2** **Pearson correlation coefficients (R) and associated *p*-values indicating the co-variation in the presence/absence of individual *mer* operons, as assessed using linear regression**. Only *mer* operons that were encoded on a chromosome were considered. Abbreviations: *merR*(c), *merR*-convergent; *merR*(d), *merR*-divergent; *merR*(a), *merR*- both convergent and divergent; Pl, plasmid encoded.

Supplementary **Figure S1** **Plot of the percent of taxa that encode for individual *mer* functions when all *mer* operons are included (chromosomal + plasmid) and the percent of taxa that encode for individual *mer* functions when only chromosomal *mer* operons are included (no plasmids)**. A 1:1 line is plotted as well. Genes that plot above the 1:1 line are suggestive of having a higher tendency to be encoded in plasmid-based *mer* operons and are more likely to be subjected to LGT than genes that plot below this line.

Supplementary **Figure S2** **The taxonomic distribution of *arsR* mapped on the MerA phylogenetic tree, as indicated by blue crosses to the right of the sequence terminal**. Phylum level taxonomic rankings are overlaid by color on each lineage.

Supplementary **Figure S3** **The taxonomic distribution of *merB* mapped on the MerA phylogenetic tree, as indicated by blue crosses to the right of the sequence terminal**. Phylum level taxonomic rankings are overlaid by color on each lineage.

Supplementary **Figure S4** **The taxonomic distribution of *merC* mapped on the MerA phylogenetic tree, as indicated by blue crosses to the right of the sequence terminal**. Phylum level taxonomic rankings are overlaid by color on each lineage.

Supplementary **Figure S5** **The taxonomic distribution of *merD* mapped on the MerA phylogenetic tree, as indicated by blue crosses to the right of the sequence terminal**. Phylum level taxonomic rankings are overlaid by color on each lineage.

Supplementary **Figure S6** **The taxonomic distribution of *merE* mapped on the MerA phylogenetic tree, as indicated by blue crosses to the right of the sequence terminal**. Phylum level taxonomic rankings are overlaid by color on each lineage.

Supplementary **Figure S7** **The taxonomic distribution of *merF* mapped on the MerA phylogenetic tree, as indicated by blue crosses to the right of the sequence terminal**. Phylum level taxonomic rankings are overlaid by color on each lineage.

Supplementary **Figure S8** **The taxonomic distribution of *merG* mapped on the MerA phylogenetic tree, as indicated by blue crosses to the right of the sequence terminal**. Phylum level taxonomic rankings are overlaid by color on each lineage.

Supplementary **Figure S9** **The taxonomic distribution of *merP* mapped on the MerA phylogenetic tree, as indicated by blue crosses to the right of the sequence terminal**. Phylum level taxonomic rankings are overlaid by color on each lineage.

Supplementary **Figure S10** **The taxonomic distribution of *merR* (divergent orientation) mapped on the MerA phylogenetic tree, as indicated by blue crosses to the right of the sequence terminal**. Phylum level taxonomic rankings are overlaid by color on each lineage.

Supplementary **Figure S11** **The taxonomic distribution of *merR* (convergent orientation) mapped on the MerA phylogenetic tree, as indicated by blue crosses to the right of the sequence terminal**. Phylum level taxonomic rankings are overlaid by color on each lineage.

Supplementary **Figure S12** **The taxonomic distribution of *merT* mapped on the MerA phylogenetic tree, as indicated by blue crosses to the right of the sequence terminal**. Phylum level taxonomic rankings are overlaid by color on each lineage.

Supplementary **Figure S13** **The taxonomic distribution of the TRASH metal binding domain protein mapped on the MerA phylogenetic tree, as indicated by blue crosses to the right of the sequence terminal**. Phylum level taxonomic rankings are overlaid by color on each lineage.

Supplementary **Figure S14** **Phylogenetic reconstruction of MerP, as determined using the Neighbor-Joining method**. The tree is rooted with a paralogous pair of proteins that are putatively involved in heavy metal transport/detoxification.

Supplementary **Figure S15** **Phylogenetic reconstruction of MerT, as determined using the Neighbor-Joining method**. The tree is rooted with a paralogous pair of proteins that are involved in binding copper.
