# Supplementary material for: The Mercury Resistance Operon: From an Origin in a Geothermal Environment to an Efficient Detoxification Machine
Source: Front Microbiol. 2012 Oct 8;3:349. doi: 10.3389/fmicb.2012.00349 (PMC3466566; doi:10.3389/fmicb.2012.00349)
Supplement: Supplementary Table S2 — Pearson correlation coefficients (R) and associated p-values indicating the co-variation in the presence/absence of individual mer operons, as assessed using linear regression. Only mer operons that were encoded on a chromosome were considered. Abbreviations: merR(c), merR-convergent; merR(d), merR-divergent; merR(a), merR- both convergent and divergent; Pl, plasmid encoded. [file 33236_Boyd_DataSheet2.DOCX]

**Supplemental Online Materials**

accompanying “The Mercury Resistance Operon: From an Origin in Geothermal Environments to an Efficient Detoxification Machine”

**Supp. Table 1 is supplied as a .zip file**

**Supp. Table 2.** Pearson correlation coefficients (R) and associated p-values indicating the co-variation in the presence/absence of individual *mer* operons, as assessed using linear regression. Only mer operons that were encoded on a chromosome were considered.

| **Pearson *R*** | *merR* (c) | *merR* (d) | *merR* (all) | *arsR* | *merP* | *merT* | *merC* | *merF* | *merE* | *merG* | *merH* | *merB* | *merD* | TRASH | Pl |
| --- | --- | --- | --- | --- | --- | --- | --- | --- | --- | --- | --- | --- | --- | --- | --- |
| *merR* (c) | **1.00** | **-0.49** | **0.32** | -0.11 | -0.03 | -0.06 | **-0.12** | -0.05 | -0.08 | 0.07 | -0.03 | -0.02 | **-0.17** | -0.06 | **-0.14** |
| *merR* (d) |  | **1.00** | **0.67** | **-0.25** | **0.48** | **0.48** | **0.28** | **0.23** | **0.34** | 0.02 | -0.06 | 0.11 | **0.40** | **-0.14** | **0.20** |
| *merR* (a) |  |  | **1.00** | **-0.36** | **0.49** | **0.47** | **0.19** | **0.21** | **0.30** | 0.09 | -0.09 | 0.10 | **0.29** | **-0.20** | 0.10 |
| *arsR* |  |  |  | **1.00** | **-0.28** | **-0.15** | **-0.15** | -0.09 | **-0.16** | -0.04 | **0.21** | **0.17** | **-0.18** | **0.46** | -0.11 |
| *merP* |  |  |  |  | **1.00** | **0.79** | **0.38** | **0.20** | **0.43** | **0.14** | -0.06 | 0.06 | **0.51** | **-0.13** | **0.19** |
| *merT* |  |  |  |  |  | **1.00** | **0.35** | **0.12** | **0.35** | **0.13** | 0.06 | **0.14** | **0.48** | **-0.14** | **0.18** |
| *merC* |  |  |  |  |  |  | **1.00** | **-0.16** | **0.39** | 0.00 | -0.03 | -0.06 | **0.57** | -0.07 | **0.17** |
| *merF* |  |  |  |  |  |  |  | **1.00** | 0.07 | -0.04 | -0.02 | -0.06 | 0.03 | -0.04 | 0.10 |
| *merE* |  |  |  |  |  |  |  |  | **1.00** | **0.12** | -0.03 | -0.01 | **0.66** | -0.07 | **0.29** |
| *merG* |  |  |  |  |  |  |  |  |  | **1.00** | -0.01 | **0.32** | **0.16** | -0.02 | **0.14** |
| *merH* |  |  |  |  |  |  |  |  |  |  | **1.00** | **0.14** | -0.04 | -0.01 | **0.12** |
| *merB* |  |  |  |  |  |  |  |  |  |  |  | **1.00** | 0.01 | -0.06 | 0.03 |
| *merD* |  |  |  |  |  |  |  |  |  |  |  |  | **1.00** | -0.08 | **0.37** |
| TRASH |  |  |  |  |  |  |  |  |  |  |  |  |  | **1.00** | -0.07 |
| Plasmid |  |  |  |  |  |  |  |  |  |  |  |  |  |  | **1.00** |
|  |  |  |  |  |  |  |  |  |  |  |  |  |  |  |  |
| ***p-*values:** | *merR* (c) | *merR* (d) | *merR* (a) | *arsR* | *merP* | *merT* | *merC* | *merF* | *merE* | *merG* | *merH* | *merB* | *merD* | TRASH | Pl |
| *merR* (c) | **<0.01** | **<0.01** | **<0.01** | 0.08 | 0.56 | 0.28 | **0.04** | 0.38 | 0.17 | 0.22 | 0.63 | 0.73 | **0.00** | 0.28 | **0.02** |
| *merR* (d) |  | **<0.01** | **<0.01** | **<0.01** | **<0.01** | **<0.01** | **<0.01** | **<0.01** | **<0.01** | 0.69 | 0.31 | 0.06 | **<0.01** | **0.02** | **0.00** |
| *merR* (a) |  |  | **<0.01** | **<0.01** | **<0.01** | **<0.01** | **0.00** | **0.00** | **<0.01** | 0.14 | 0.13 | 0.08 | **<0.01** | **0.00** | 0.09 |
| *arsR* |  |  |  | **<0.01** | **<0.01** | **0.01** | **0.01** | 0.13 | **0.01** | 0.51 | **0.00** | **0.00** | **0.00** | **< 0.001** | 0.07 |
| *merP* |  |  |  |  | **<0.01** | **<0.01** | **<0.01** | **0.00** | **<0.01** | **0.02** | 0.34 | 0.33 | **<0.01** | **0.03** | **0.00** |
| *merT* |  |  |  |  |  | **<0.01** | **<0.01** | **0.04** | **<0.01** | **0.03** | 0.35 | **0.02** | **<0.01** | **0.02** | **0.00** |
| *merC* |  |  |  |  |  |  | **<0.01** | **0.01** | **<0.01** | 1.00 | 0.62 | 0.32 | **<0.01** | 0.26 | **0.00** |
| *merF* |  |  |  |  |  |  |  | **<0.01** | 0.23 | 0.48 | 0.75 | 0.31 | 0.63 | 0.48 | 0.08 |
| *merE* |  |  |  |  |  |  |  |  | **<0.01** | **0.04** | 0.59 | 0.86 | **<0.01** | 0.23 | **<0.01** |
| *merG* |  |  |  |  |  |  |  |  |  | **<0.01** | 0.89 | **<0.01** | **0.01** | 0.76 | **0.02** |
| *merH* |  |  |  |  |  |  |  |  |  |  | **<0.01** | **0.02** | 0.54 | 0.89 | **0.04** |
| *merB* |  |  |  |  |  |  |  |  |  |  |  | **<0.01** | 0.91 | 0.34 | 0.66 |
| *merD* |  |  |  |  |  |  |  |  |  |  |  |  | **<0.01** | 0.16 | **<0.01** |
| TRASH |  |  |  |  |  |  |  |  |  |  |  |  |  | **<0.01** | 0.28 |
| Plasmid |  |  |  |  |  |  |  |  |  |  |  |  |  |  | **<0.01** |

Abbreviations: merR(c), merR-convergent; merR(d), merR-divergent; merR(a), merR- both convergent and divergent; Pl, plasmid encoded.


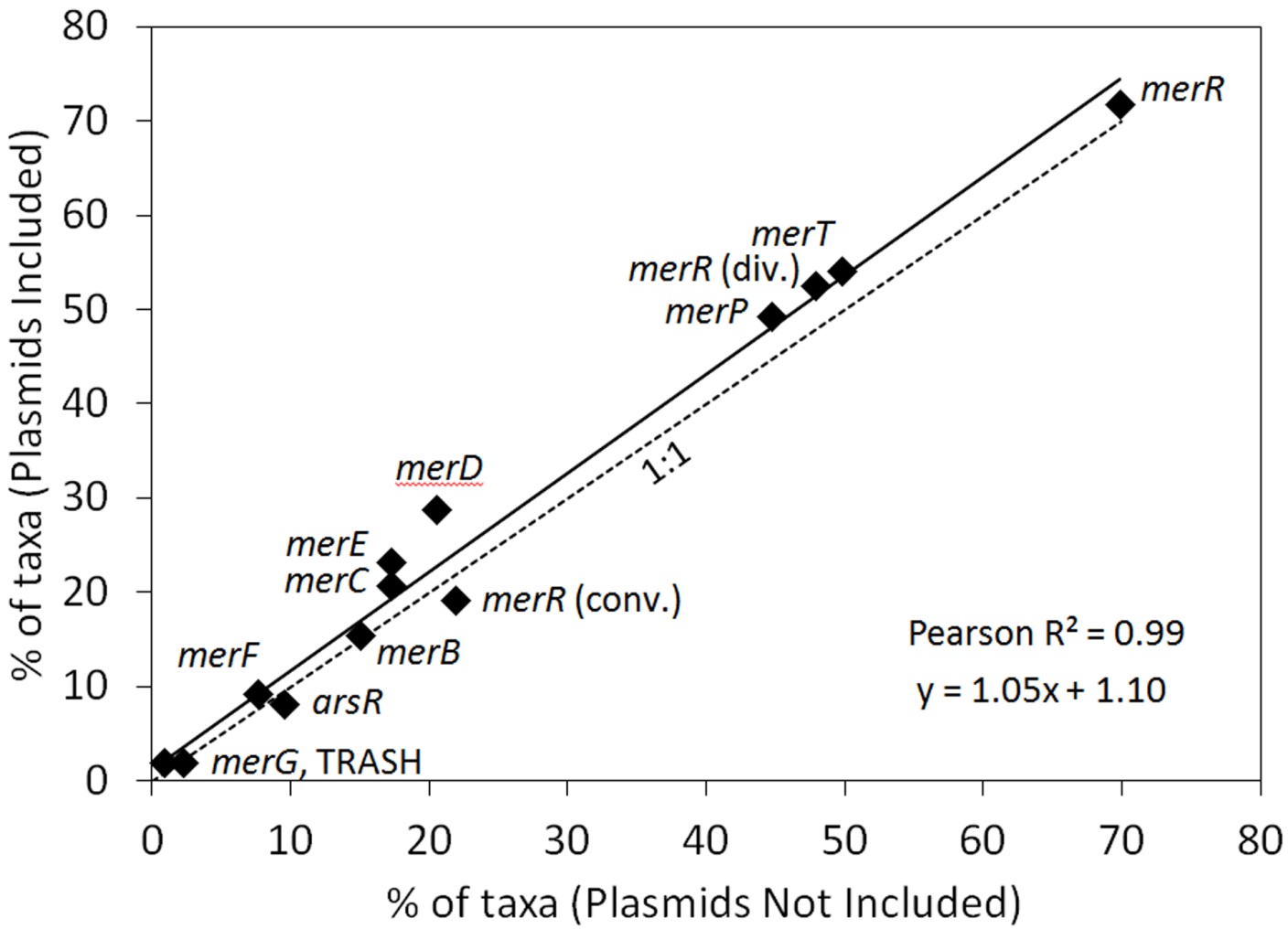


**Supp. Figure. 1.** Plot of the percent of taxa that encode for individual *mer* functions when all *mer* operons are included (chromosomal + plasmid) and the percent of taxa that encode for individual *mer* functions when only chromosomal *mer* operons are included (no plasmids). A 1:1 line is plotted as well. Genes that plot above the 1:1 line are suggestive of having a higher tendency to be encoded in plasmid-based mer operons and are more likely to be subjected to LGT than genes that plot below this line.


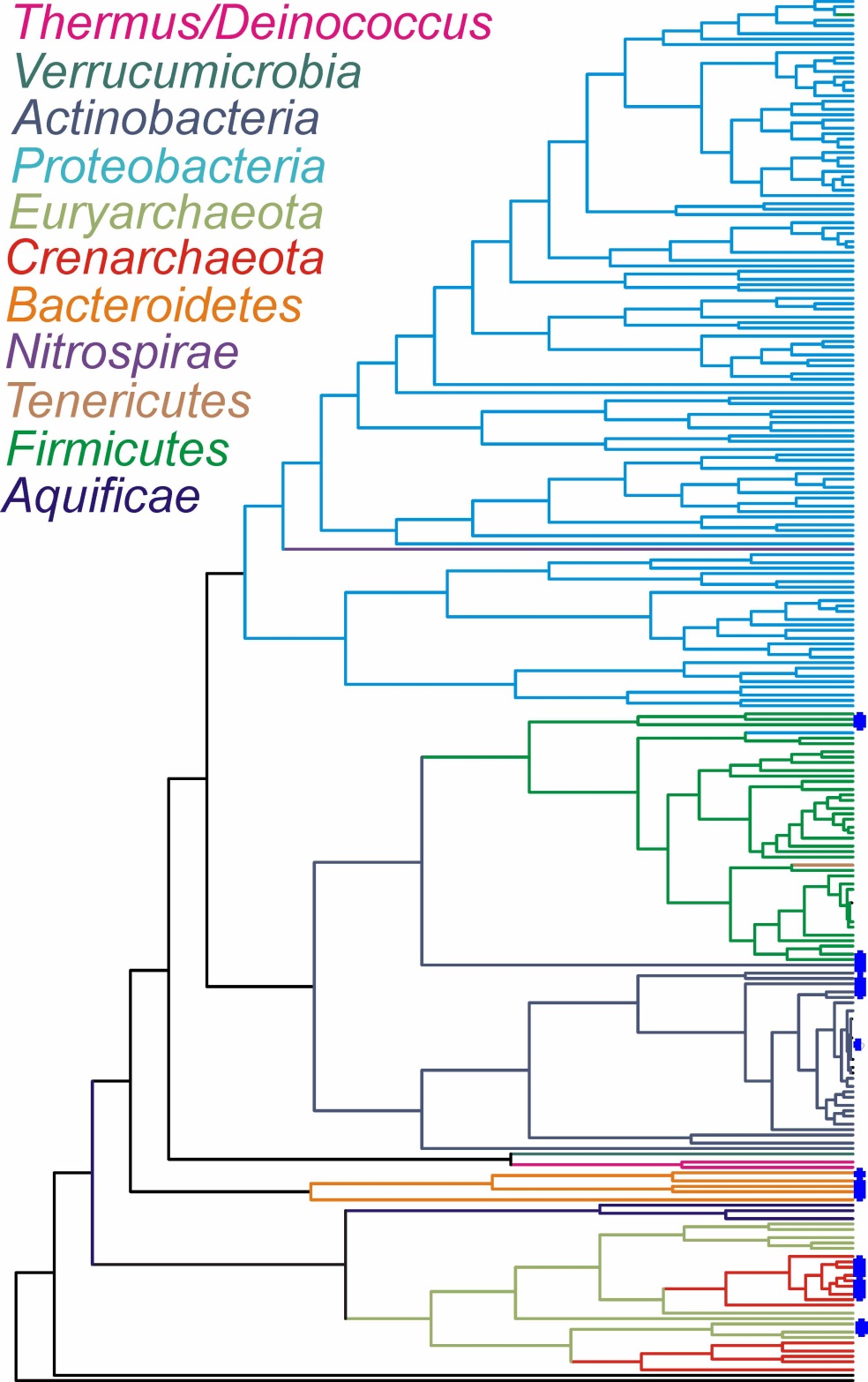


**Supp. Figure 2.** The taxonomic distribution of *arsR* mapped on the MerA phylogenetic tree, as indicated by blue crosses to the right of the sequence terminal. Phylum level taxonomic rankings are overlaid by color on each lineage.


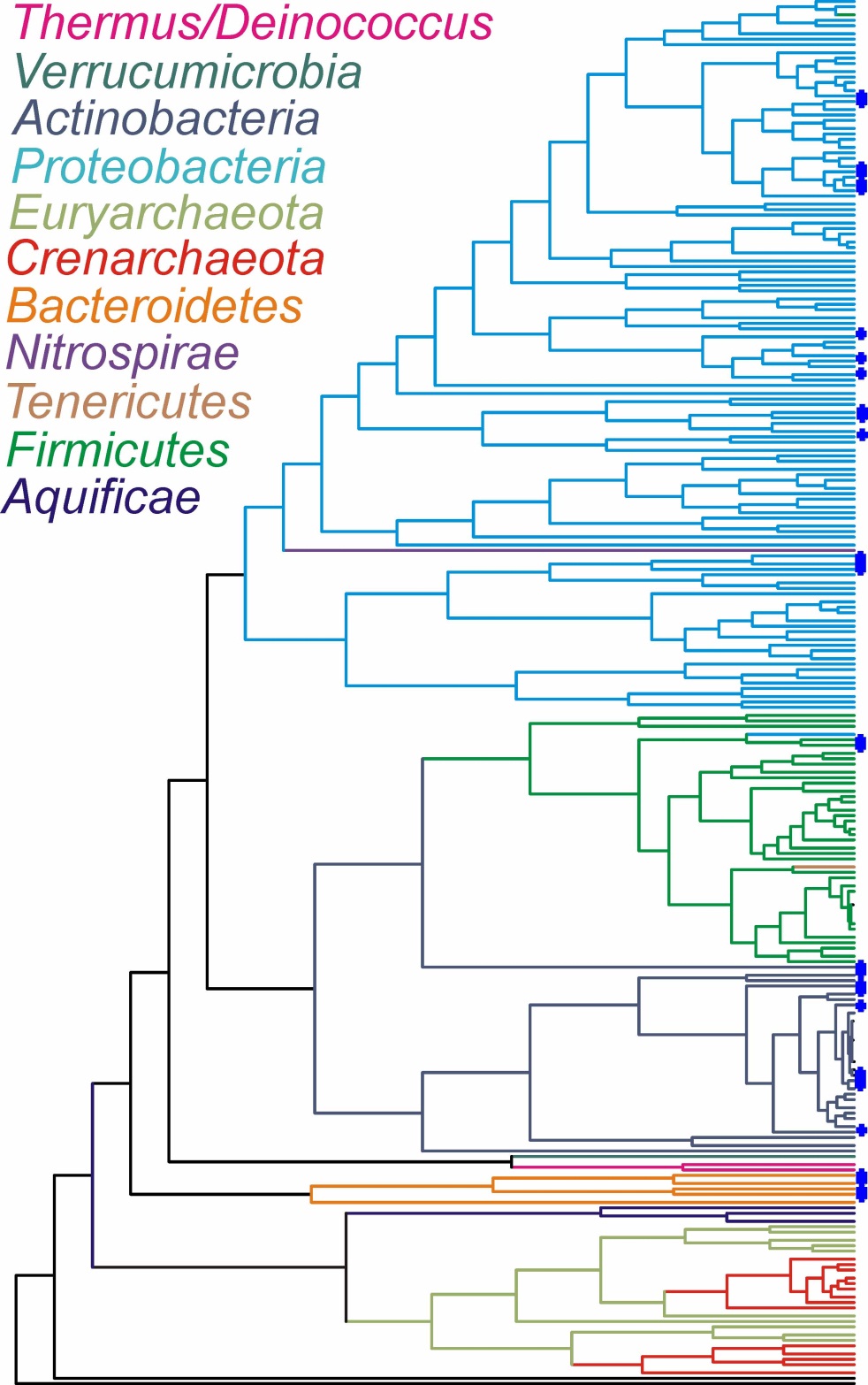


**Supp. Figure 3.** The taxonomic distribution of *merB* mapped on the MerA phylogenetic tree, as indicated by blue crosses to the right of the sequence terminal. Phylum level taxonomic rankings are overlaid by color on each lineage.


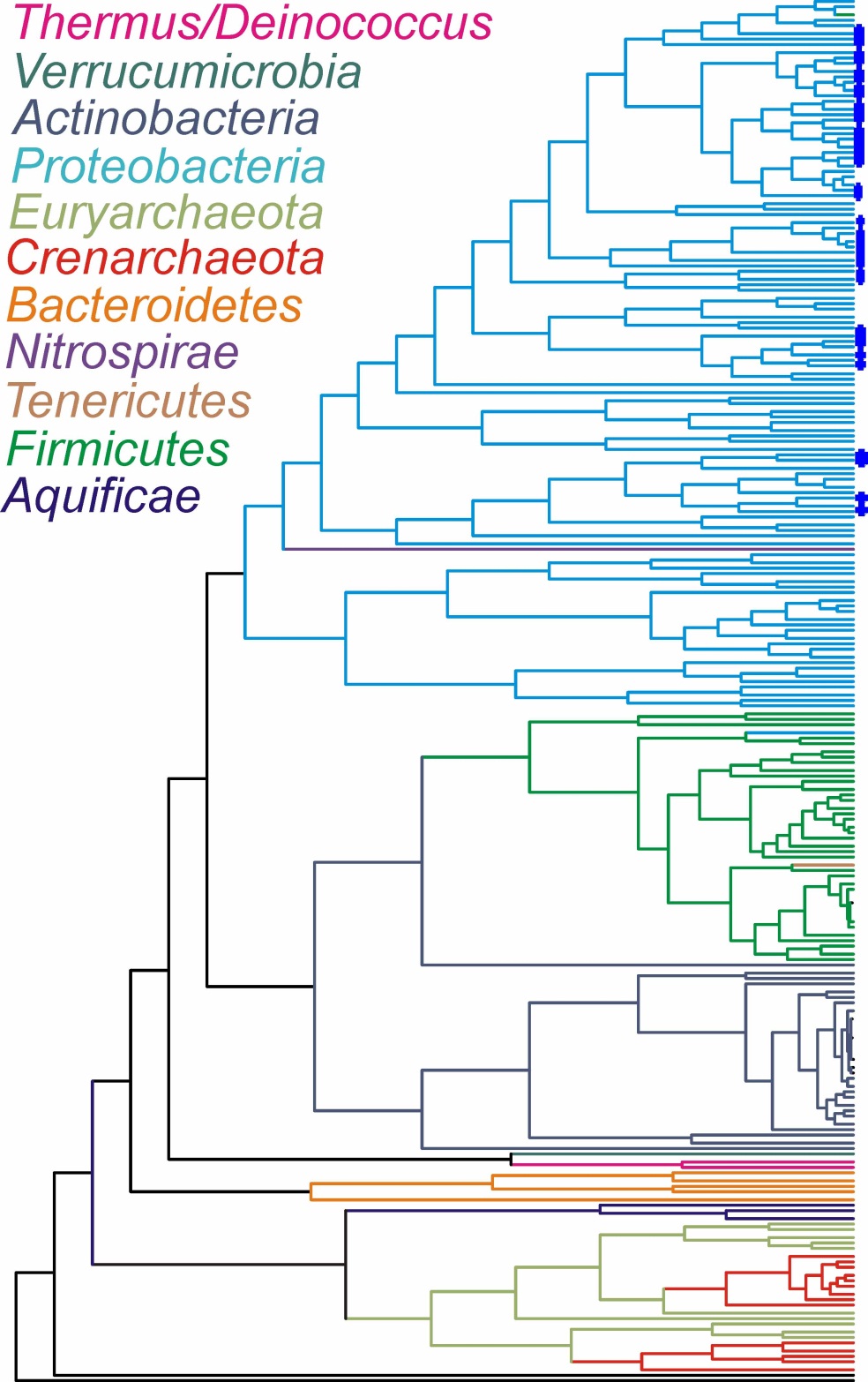


**Supp. Figure 4.** The taxonomic distribution of *merC* mapped on the MerA phylogenetic tree, as indicated by blue crosses to the right of the sequence terminal. Phylum level taxonomic rankings are overlaid by color on each lineage.


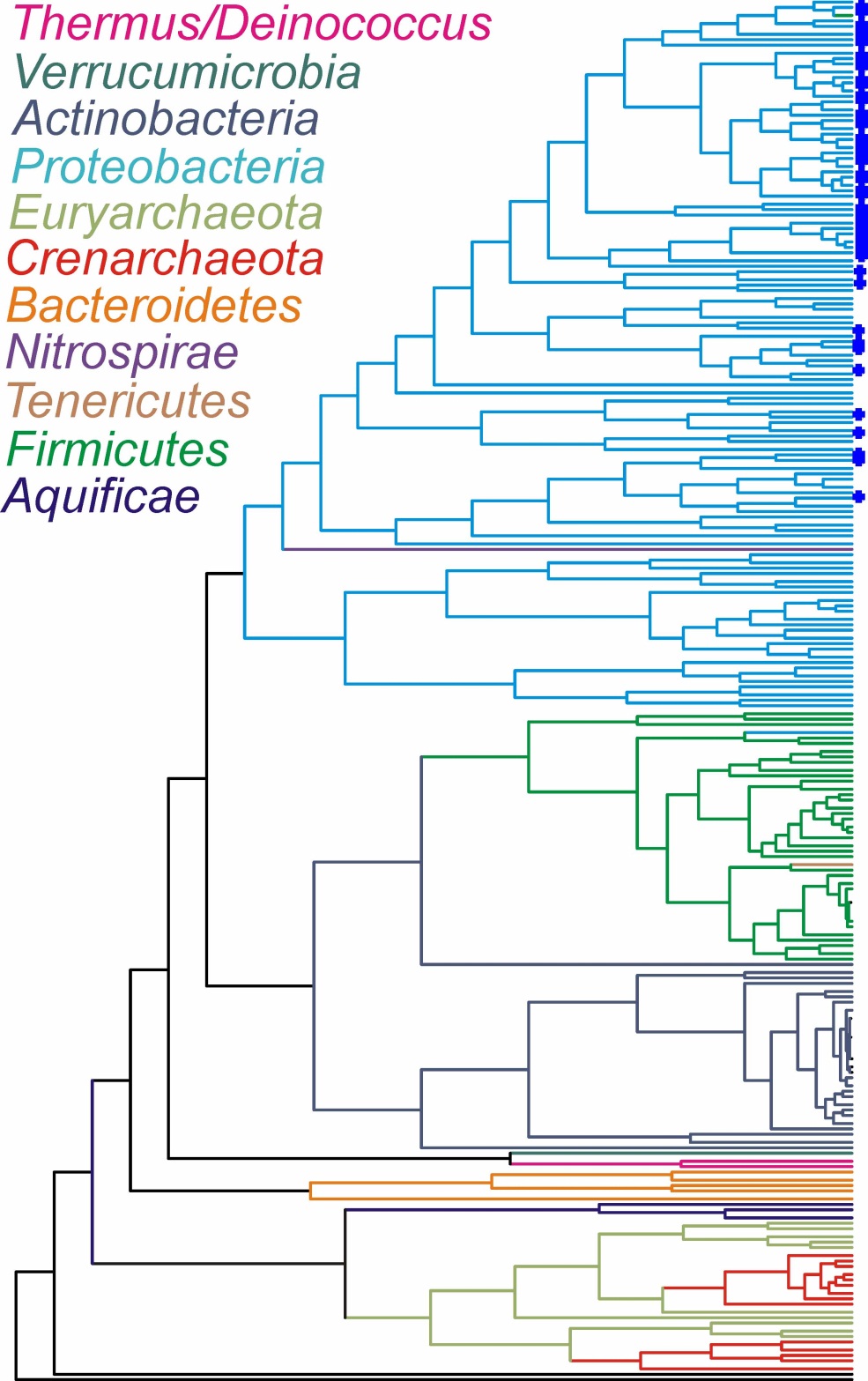


**Supp. Figure 5.** The taxonomic distribution of *merD* mapped on the MerA phylogenetic tree, as indicated by blue crosses to the right of the sequence terminal. Phylum level taxonomic rankings are overlaid by color on each lineage.


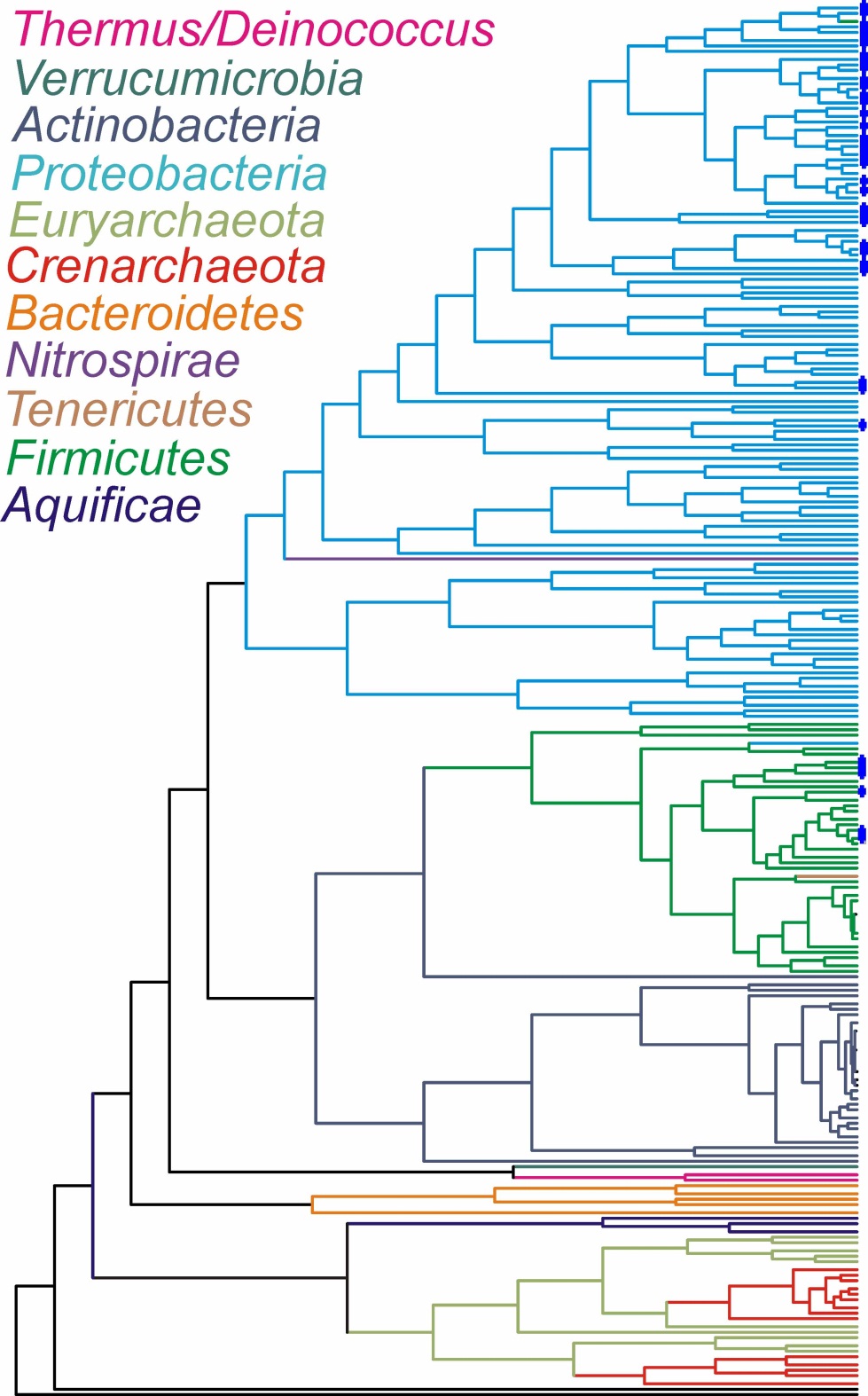


**Supp. Figure 6.** The taxonomic distribution of *merE* mapped on the MerA phylogenetic tree, as indicated by blue crosses to the right of the sequence terminal. Phylum level taxonomic rankings are overlaid by color on each lineage.


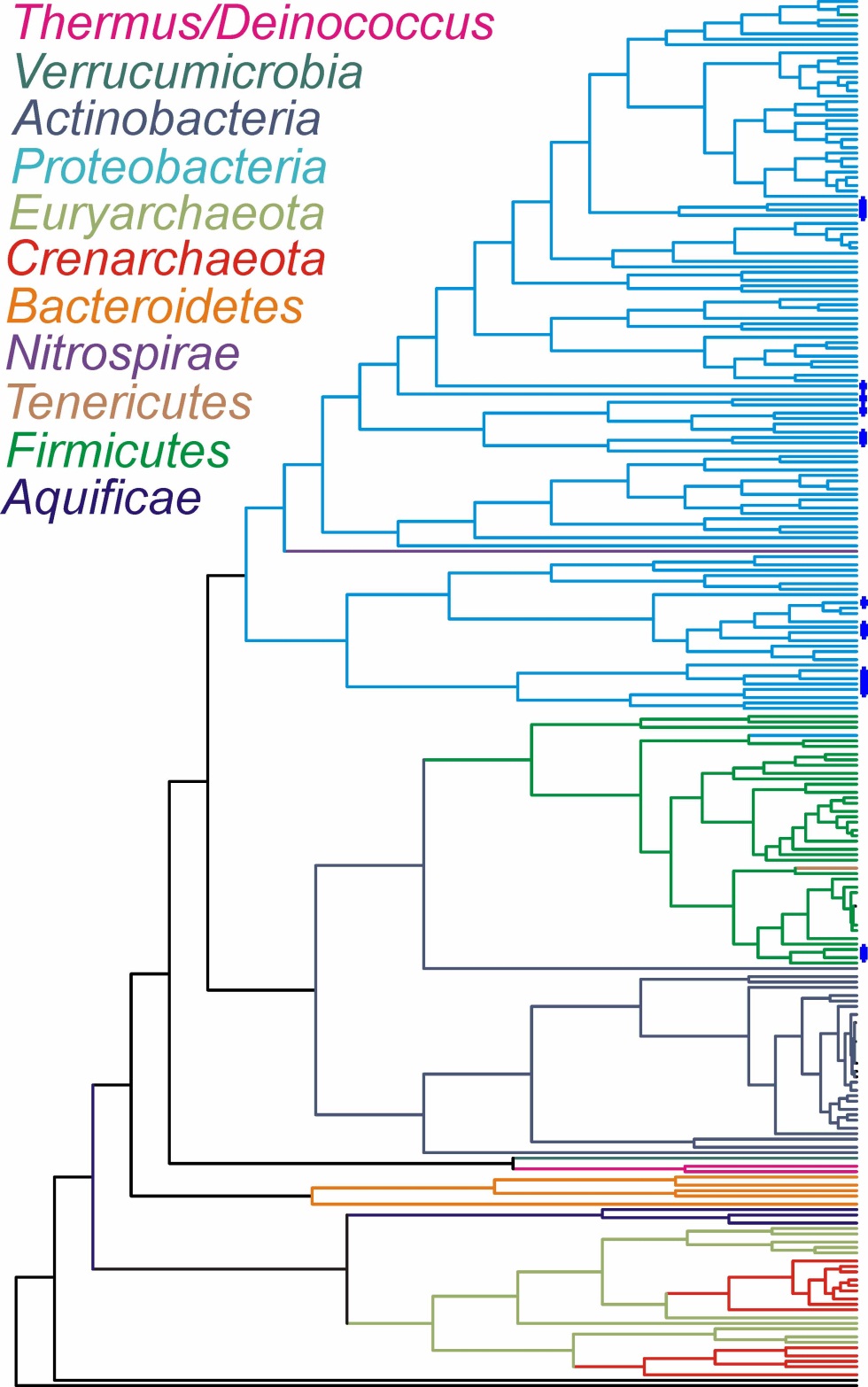


**Supp. Figure 7.** The taxonomic distribution of *merF* mapped on the MerA phylogenetic tree, as indicated by blue crosses to the right of the sequence terminal. Phylum level taxonomic rankings are overlaid by color on each lineage.


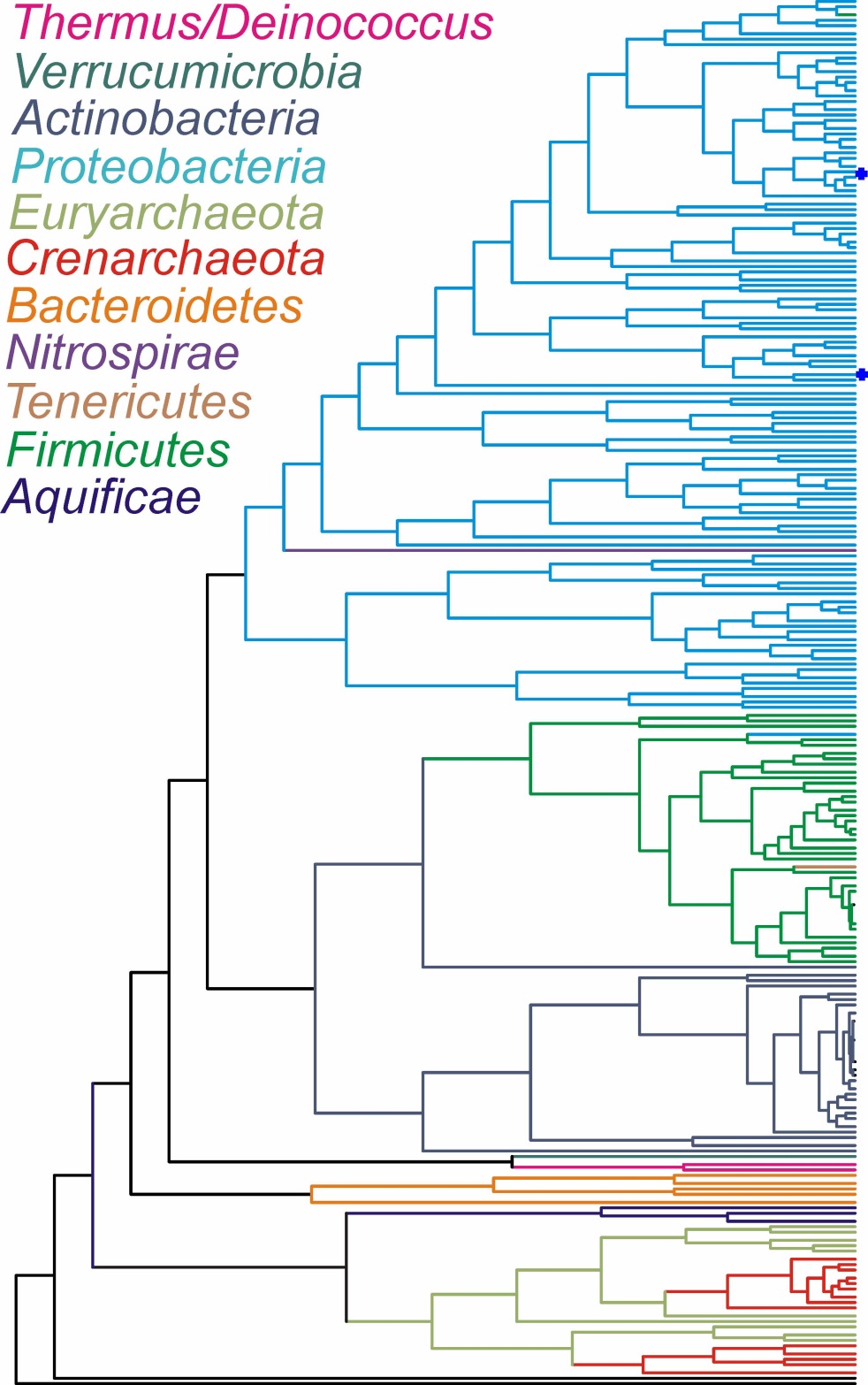


**Supp. Figure 8.** The taxonomic distribution of *merG* mapped on the MerA phylogenetic tree, as indicated by blue crosses to the right of the sequence terminal. Phylum level taxonomic rankings are overlaid by color on each lineage.


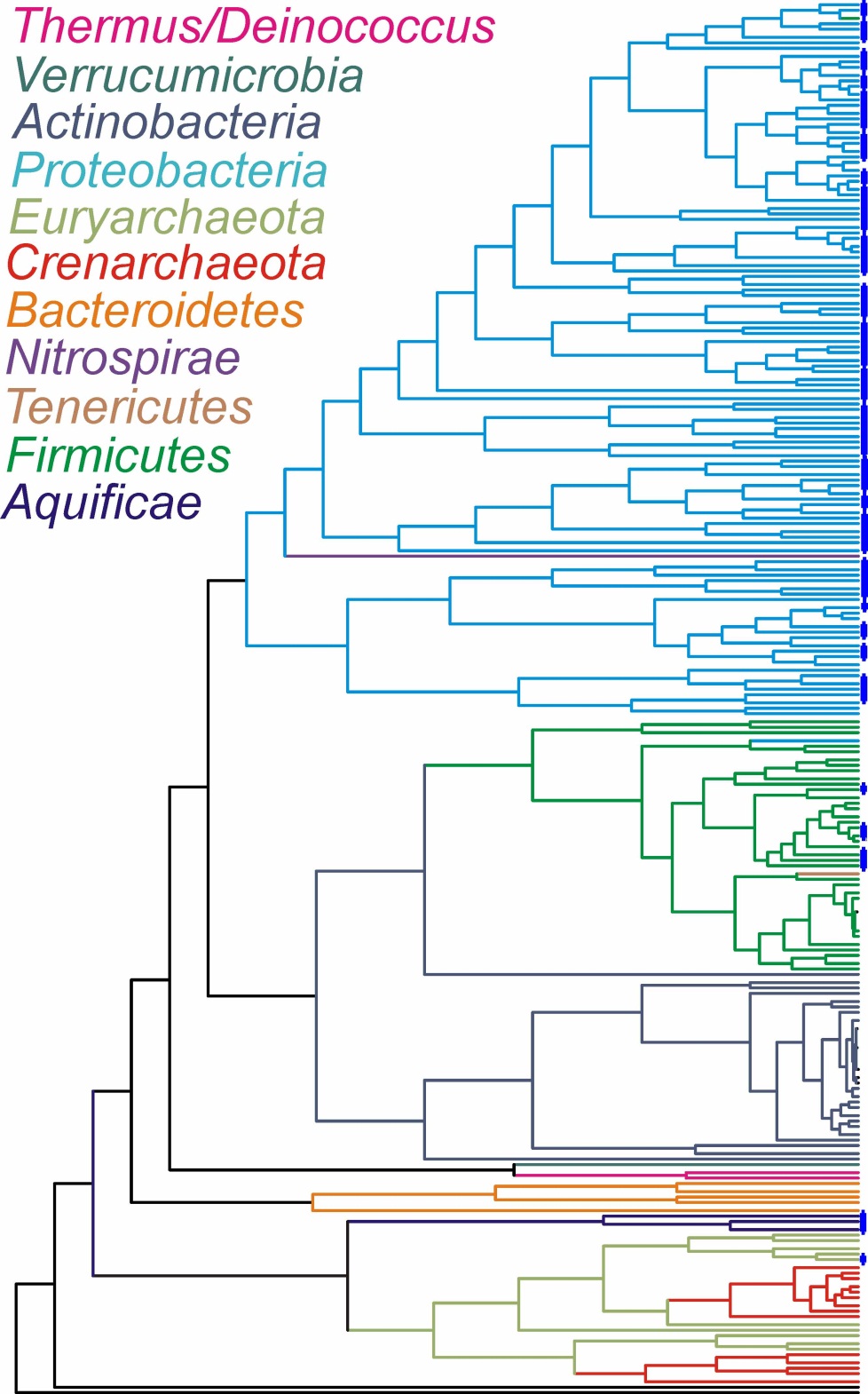


**Supp. Figure 9.** The taxonomic distribution of *merP* mapped on the MerA phylogenetic tree, as indicated by blue crosses to the right of the sequence terminal. Phylum level taxonomic rankings are overlaid by color on each lineage.


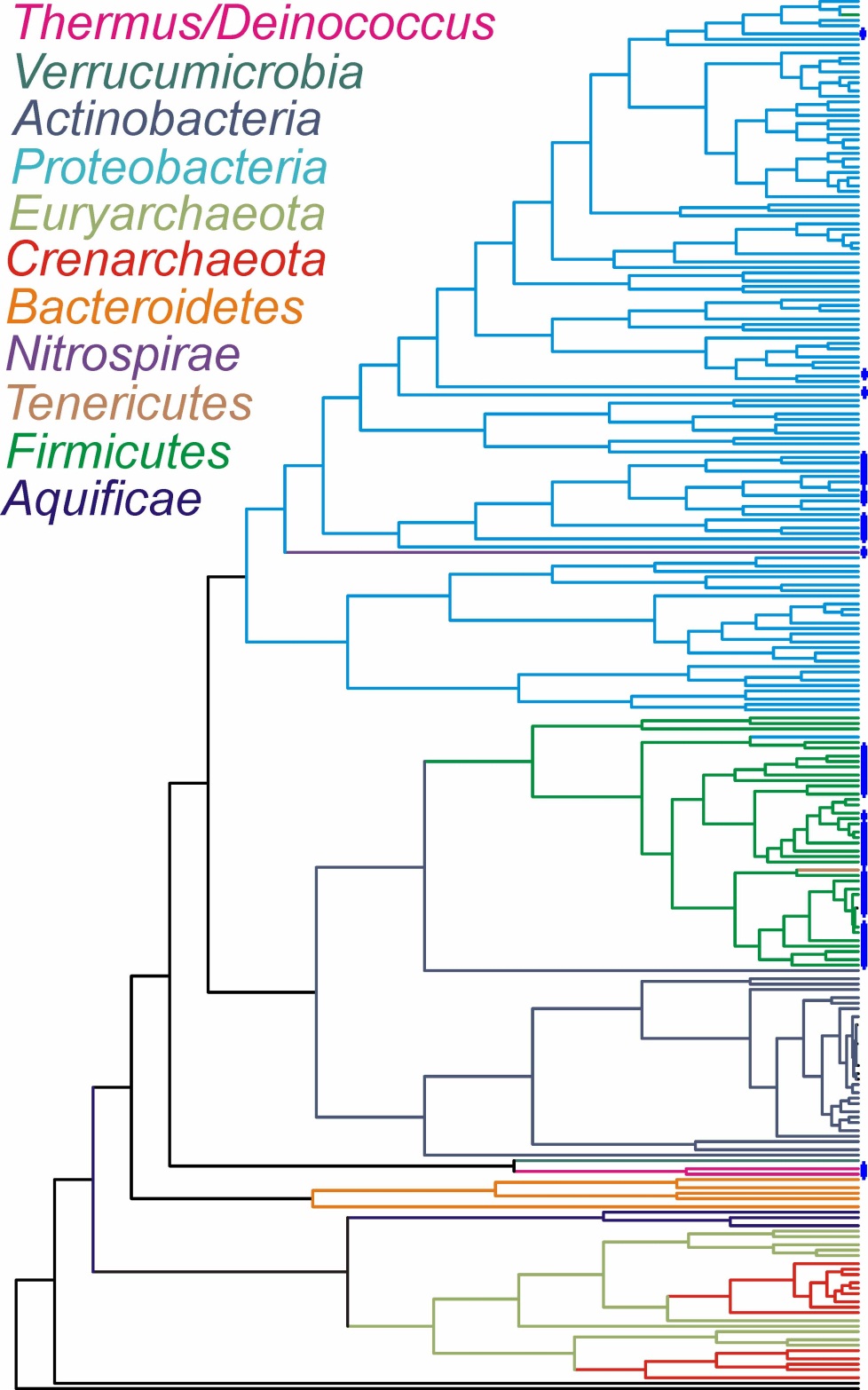


**Supp. Figure 10.** The taxonomic distribution of *merR* (divergent orientation) mapped on the MerA phylogenetic tree, as indicated by blue crosses to the right of the sequence terminal. Phylum level taxonomic rankings are overlaid by color on each lineage.


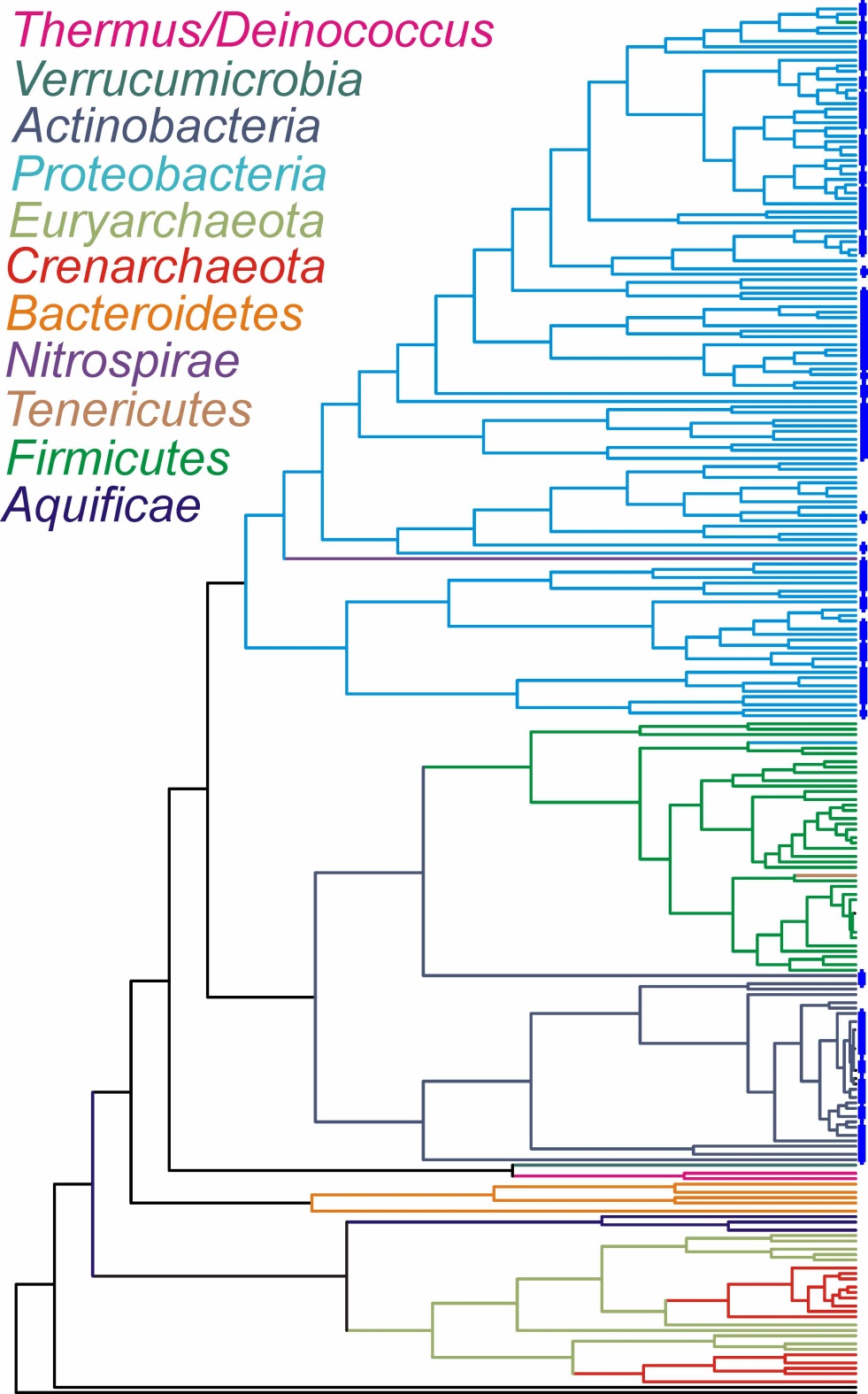


**Supp. Figure 11.** The taxonomic distribution of *merR* (convergent orientation) mapped on the MerA phylogenetic tree, as indicated by blue crosses to the right of the sequence terminal. Phylum level taxonomic rankings are overlaid by color on each lineage.


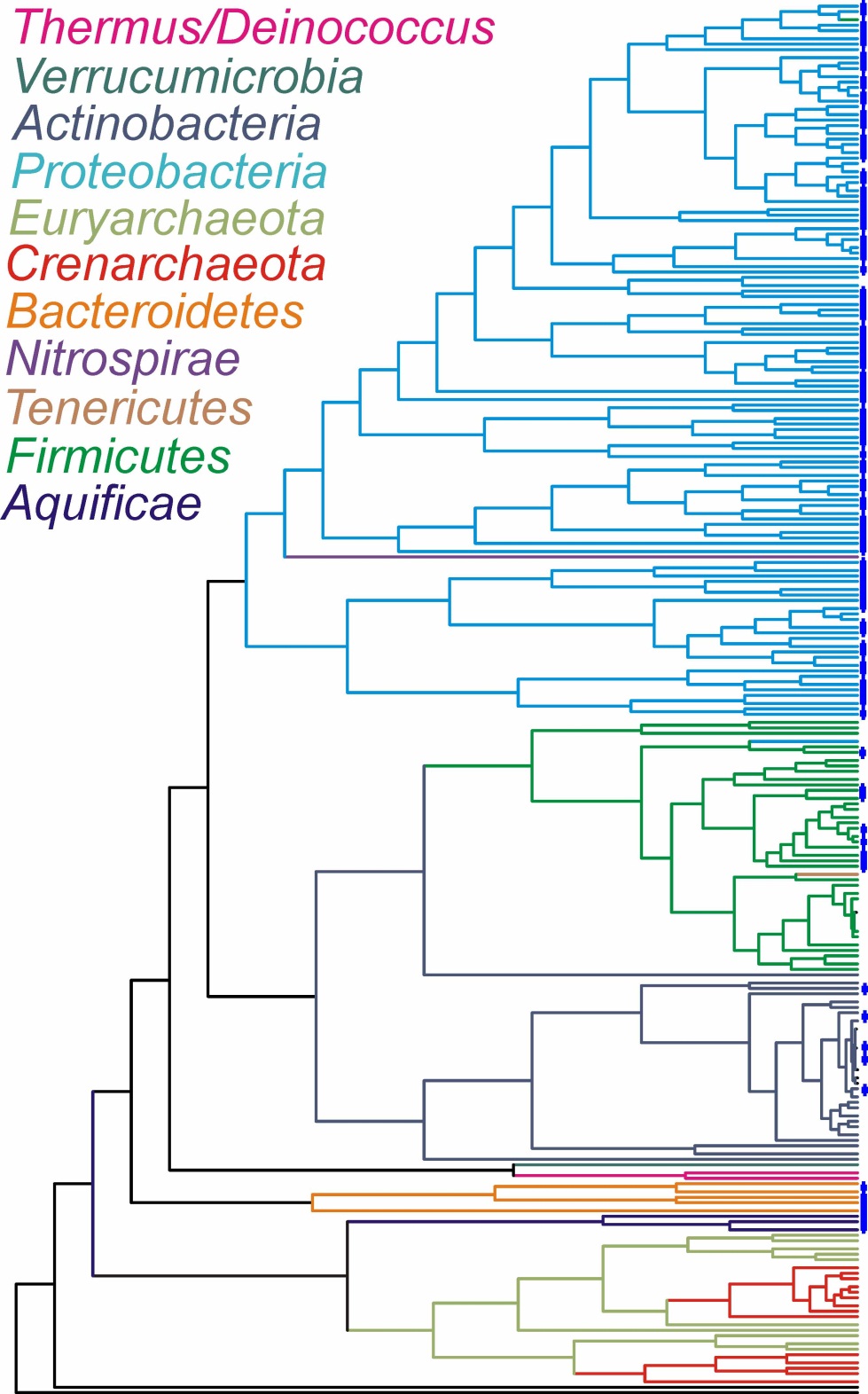


**Supp. Figure 12.** The taxonomic distribution of *merT* mapped on the MerA phylogenetic tree, as indicated by blue crosses to the right of the sequence terminal. Phylum level taxonomic rankings are overlaid by color on each lineage.


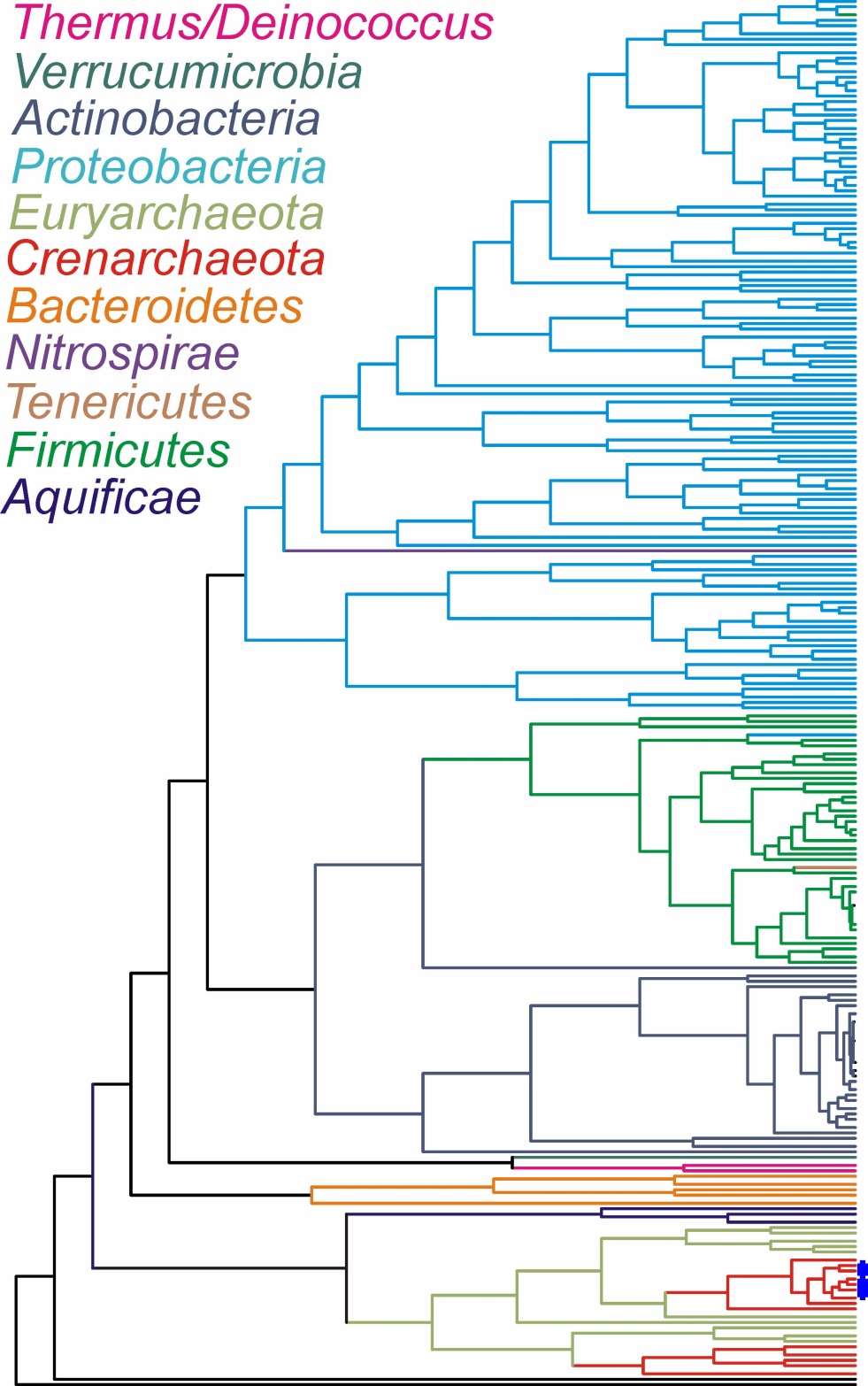


**Supp. Figure 13.** The taxonomic distribution of the TRASH metal binding domain protein mapped on the MerA phylogenetic tree, as indicated by blue crosses to the right of the sequence terminal. Phylum level taxonomic rankings are overlaid by color on each lineage.


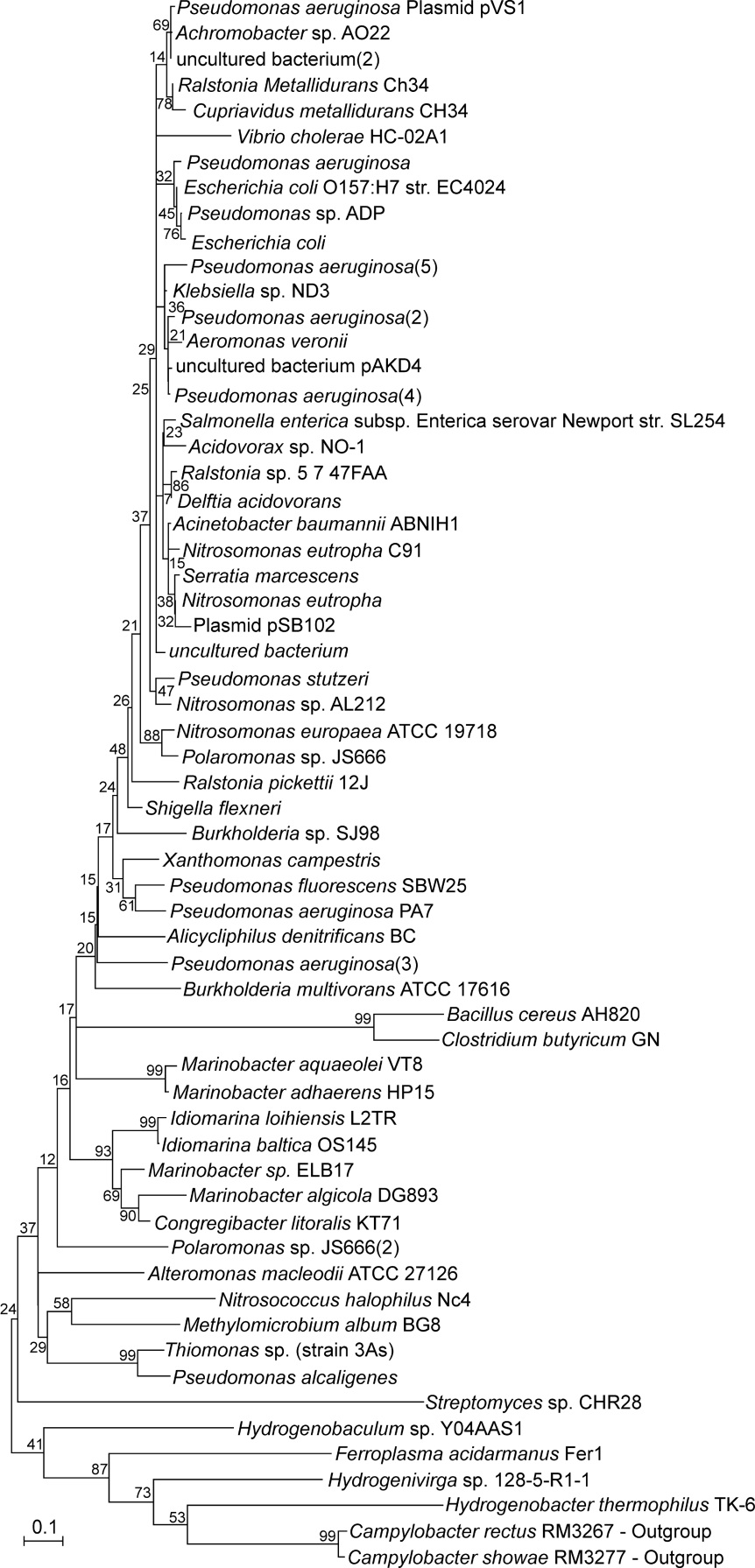


**Supp. Figure 14.** Phylogenetic reconstruction of MerP, as determined using the Neighbor-Joining method. The tree is rooted with a paralogous pair of proteins that are putatively involved in heavy metal transport/detoxification.


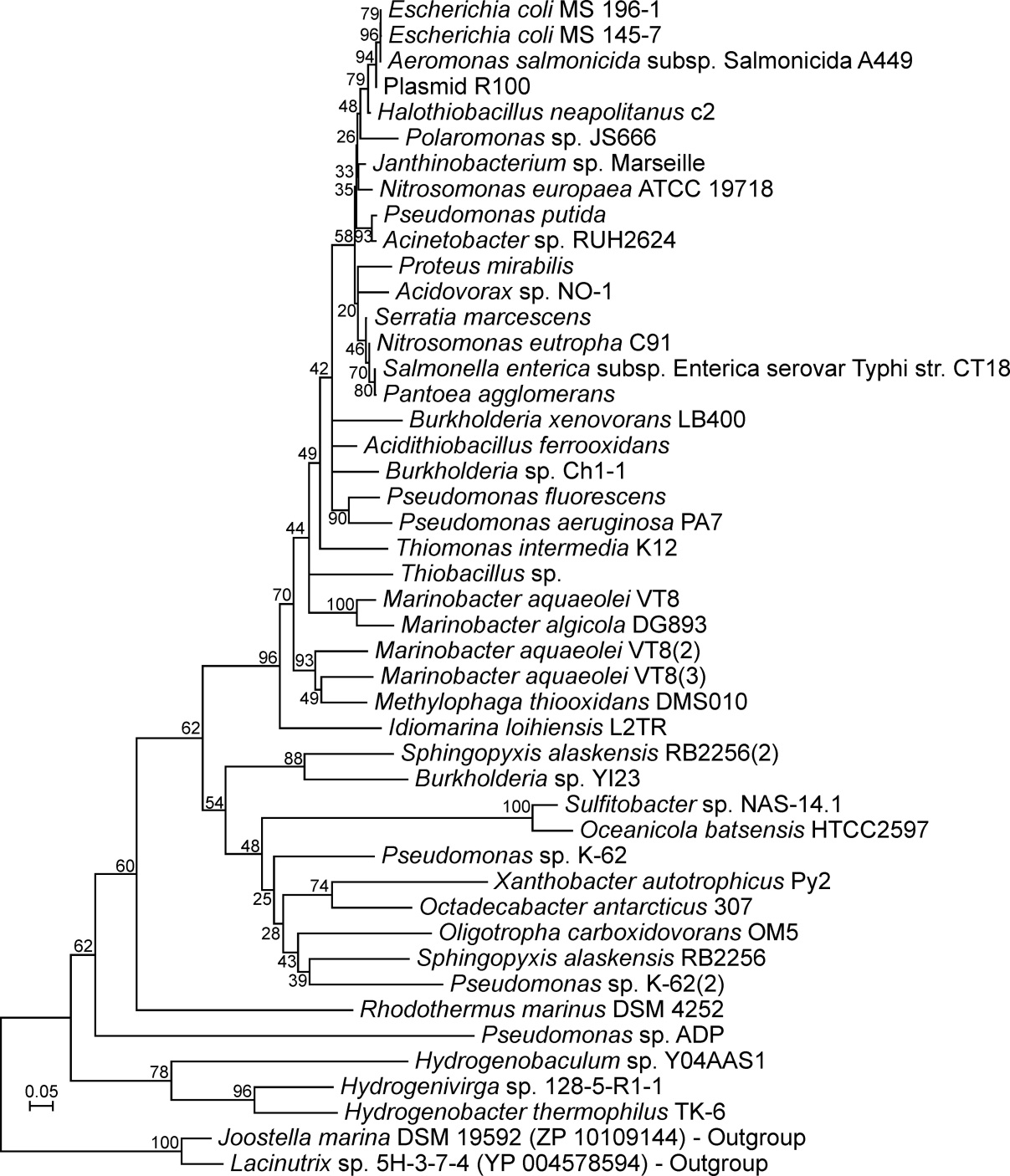


**Supp. Figure 15.** Phylogenetic reconstruction of MerT, as determined using the Neighbor-Joining method. The tree is rooted with a paralogous pair of proteins that are involved in binding copper.
